# Cognitive and neuropsychiatric effects of noradrenergic treatment in Alzheimer’s disease: systematic review and meta-analysis

**DOI:** 10.1136/jnnp-2022-329136

**Published:** 2022-07-05

**Authors:** Michael C B David, Martina Del Giovane, Kathy Y Liu, Benjamin Gostick, James Benedict Rowe, Imafidon Oboh, Robert Howard, Paresh A Malhotra

**Affiliations:** 1 Imperial College London and the University of Surrey, UK Dementia Research Institute Care Research and Technology Centre, London, UK; 2 Brain Sciences, Imperial College London, London, UK; 3 Imperial College Healthcare NHS Trust, Clinical Neurosciences, Charing Cross Hospital, London, UK; 4 Division of Psychiatry, University College London, London, UK; 5 Department of Clinical Neurosciences, University of Cambridge, Cambridge, UK; 6 South West London and St George’s Mental Health NHS Trust, London, UK

**Keywords:** Alzheimer's disease, apathy, cognition, dementia, psychiatry

## Abstract

**Background:**

Dysfunction of the locus coeruleus-noradrenergic system occurs early in Alzheimer’s disease, contributing to cognitive and neuropsychiatric symptoms in some patients. This system offers a potential therapeutic target, although noradrenergic treatments are not currently used in clinical practice.

**Objective:**

To assess the efficacy of drugs with principally noradrenergic action in improving cognitive and neuropsychiatric symptoms in Alzheimer’s disease.

**Methods:**

The MEDLINE, Embase and ClinicalTrials.gov databases were searched from 1980 to December 2021. We generated pooled estimates using random effects meta-analyses.

**Results:**

We included 19 randomised controlled trials (1811 patients), of which six were judged as ‘good’ quality, seven as ‘fair’ and six ‘poor’. Meta-analysis of 10 of these studies (1300 patients) showed a significant small positive effect of noradrenergic drugs on global cognition, measured using the Mini-Mental State Examination or Alzheimer’s Disease Assessment Scale—Cognitive Subscale (standardised mean difference (SMD): 0.14, 95% CI: 0.03 to 0.25, p=0.01; I^2^=0%). No significant effect was seen on measures of attention (SMD: 0.01, 95% CI: −0.17 to 0.19, p=0.91; I^2^=0). The apathy meta-analysis included eight trials (425 patients) and detected a large positive effect of noradrenergic drugs (SMD: 0.45, 95% CI: 0.16 to 0.73, p=0.002; I^2^=58%). This positive effect was still present following removal of outliers to account for heterogeneity across studies.

**Discussion:**

Repurposing of established noradrenergic drugs is most likely to offer effective treatment in Alzheimer’s disease for general cognition and apathy. However, several factors should be considered before designing future clinical trials. These include targeting of appropriate patient subgroups and understanding the dose effects of individual drugs and their interactions with other treatments to minimise risks and maximise therapeutic effects.

**PROSPERO registeration number:**

CRD42021277500.

**video abstract:** Disclaimer: this video summarises a scientific article published by BMJ Publishing Group Limited (BMJ). The content of this video has not been peer-reviewed and does not constitute medical advice. Any opinions expressed are solely those of the contributors. Viewers should be aware that professionals in the field may have different opinions. BMJ does not endorse any opinions expressed or recommendations discussed. Viewers should not use the content of the video as the basis for any medical treatment. BMJ disclaims all liability and responsibility arising from any reliance placed on the content.

**WHAT IS ALREADY KNOWN ON THIS TOPIC**

- Noradrenergic dysfunction occurs early in Alzheimer’s disease and contributes to cognitive and neuropsychiatric symptoms.

**WHAT THIS STUDY ADDS**

- There is good evidence that drugs with principally noradrenergic action could be effective in treating cognitive symptoms and apathy in Alzheimer’s disease.

**HOW THIS STUDY MIGHT AFFECT RESEARCH, PRACTICE AND/OR POLICY**

- There is a strong rationale for further, targeted clinical trials of noradrenergic treatments in Alzheimer’s disease.

## Introduction

The neurotransmitter noradrenaline (NA), also called norepinephrine, is critical for arousal and many cognitive processes including attention, learning, memory, executive and inhibitory control.^1–3^ It is predominantly synthesised and released by specialised noradrenergic neurons originating from the locus coeruleus (LC).^4^ Diffuse projections throughout the cerebrum act on three main adrenoreceptor (AR) classes^5^: α_1_, α_2_ and β. Generally, stimulation of α_1_-AR and β-ARs enhances neurotransmission and plasticity whereas presynaptic α_2_-ARs autoinhibit NA release.^5^ In addition to neurotransmission, NA regulates microglial surveillance and synaptotoxicity.^6^

The progression of tau pathology in Alzheimer’s disease may begin in the LC, where neuronal loss occurs early in the disease.^7 8^ In vivo studies using neuromelanin-sensitive MRI show LC degeneration in Alzheimer’s disease compared with healthy ageing.^9^ The noradrenergic system’s role in attention, memory and executive functions makes the loss of LC noradrenergic cells of immediate relevance to Alzheimer’s dementia.^10 11^ The LC-NA system is also related to behavioural and neuropsychiatric symptoms in Alzheimer’s disease.^12^ For example, apathy is common in Alzheimer’s disease,^13^ and motivation is influenced by the noradrenergic system.^3^ However, current symptomatic treatments of Alzheimer’s disease focus on restoration of cholinergic and glutamatergic systems, with only modest effects.^14^ Given the early changes in the LC-NA system in Alzheimer’s disease, it is a potential target for treatments of cognitive and behavioural dysfunction.^10 15^

A range of drugs act via noradrenergic pathways. Synaptic availability of NA is increased by inhibition of reuptake and degradation, while receptors can be directly stimulated/blocked.^16^ Animal and human studies indicate the potential therapeutic use of drugs that act on all three receptor classes.^5 17–19^ For example, post-synaptic α_2A_-ARs regulate prefrontal cortex dependent cognition and their agonism by guanfacine can improve cognition and prefrontal cortical network connectivity.^18 20^ Many drugs in clinical use inhibit reuptake of NA, preventing synaptic clearance.^16^ Examples include selective noradrenergic reuptake inhibitors (NRIs) and less selective serotonin-norepinephrine reuptake inhibitors, known as antidepressants but also used for anxiety, pain and neuropathy. Established treatments for attention deficit hyperactivity disorder, including methylphenidate^21^ and atomoxetine,^22^ work via this mechanism. Modafinil, a stimulant, acts partly through NA reuptake inhibition. Clinical trials of noradrenergic treatments in neurodegenerative conditions were first undertaken decades ago.^23–25^ However, after a series of small studies failed to provide convincing evidence for treatment benefits in Alzheimer’s disease, interest waned. Recently, recognition of early LC-NA involvement in Alzheimer’s disease has increased alongside new tools to assay the system in vivo,^26 27^ and new drug options.

We therefore performed a systematic review and meta-analysis of drugs with principally noradrenergic action in Alzheimer’s disease. We acknowledge that the drugs included here are not purely noradrenergic in their action but at the doses used, this is likely to have been their primary mechanism. We assessed the evidence for the extent to which noradrenergic agents show therapeutic benefit, on cognitive and behavioural aspects of Alzheimer’s disease.

## Methods

The protocol was registered with the International Prospective Register of Systematic Reviews. We conducted and reported the study in accordance with Preferred Reporting Items for Systematic reviews and Meta-Analyses guidelines (online supplemental table S3).^28^

10.1136/jnnp-2022-329136.supp1 Supplementary data

### Eligibility criteria

**Figure 1:**
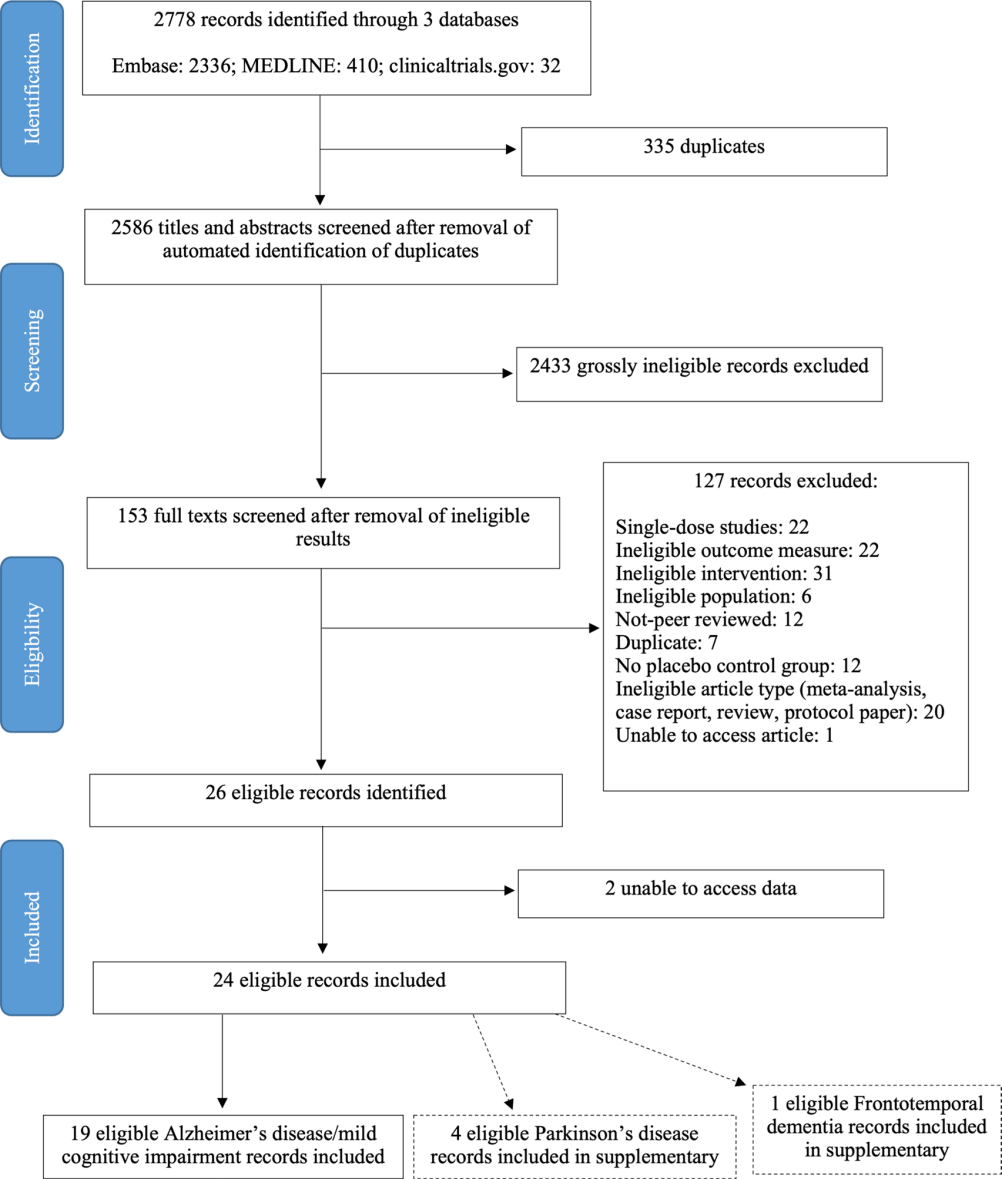
Preferred Reporting Items for Systematic Reviews and Meta-Analyses flow diagram for search for studies reporting the use of noradrenergic therapies in neurodegenerative conditions. Some records excluded for more than one reason.

We searched MEDLINE, Embase and ClinicalTrials.gov for studies that fulfilled all criteria: (1) study populations defined as patients with any of Alzheimer’s disease, mild cognitive impairment (MCI), Parkinson’s disease (PD), Lewy Body dementia, frontotemporal dementia (FTD) or progressive supranuclear palsy; (2) prospective clinical trials that compared drugs that increase the level of NA or act on one of the three receptor classes, versus placebo and (3) studies that reported cognitive, neuropsychiatric or behavioural outcomes (table 1). Flow diagram of search is shown in figure 1. Full search terms and inclusion/exclusion criteria are listed in table 2. Ongoing clinical trials of noradrenergic agents were not included.

**Table 1:** Included Alzheimer’s disease studies

Study (a/b/c=separate study arms)		Participants				Intervention				Outcomes included
		% Female	Mean age, years	n						
				Drug	Placebo	Class	Drug	Daily dosage, mg	Duration, weeks	
Herrmann 2008*^66^		53.8	77.9	13	13	NRI	Methylphenidate	20	2	1a, 9a
Lanctot 2014†^67^		61.6	76	29	31	NRI	Methylphenidate	20	6	1a, 2a, 7a
Rosenberg 2013†^68^		61.6	76	29	31	NRI	Methylphenidate	20	6	9a
Maier 2020^69^		38	74.8	54	54	NRI	Buproprion	150–300	12	1a, 3a, 8a
Mintzer 2021^13^		33	76	101	99	NRI	Methylphenidate	20	26	1a, 2a, 9b, 4a, 6a, 7a, 10a
Padala 2018^45^		0	76.6	30	30	NRI	Methylphenidate	10–20	12	1a, 9a
Mohs 2009^22^		54.3	77.4	47	45	NRI	Atomoxetine	25–80	26	1a
Frakey 2012^46^		Unknown	77.3	11	11	NRI	Modafinil	200	8	9c
Levey 2021*‡^70^		46.2	70.3	39	39	NRI	Atomoxetine	100	26	1a, 2c, 4c, 6e, 7d, 8a, 9b, 10a
Winblad 2001^71^		62.4	73.7	177	169	A1 Ant	Nicergoline	60	26	1b
Amaducci 1999^72^		Unknown	Unknown	102	95	A1 Ant	Nicergoline	60	52	1b
Banerjee 2021^73^		66	82.8	102	102	A1 Ant	Mirtazapine	45	12	8a, 10b
Wang 2009^74^		40.9	80.6	11	11	A1 Ant	Prazosin	6	8	8a
Crook 1992^25^		55	71	15	14	A2 Ag	Guanfacine	0.5	13	3a, 5a, 7b
Mohr 1989*^23^	a	25	62	8	8	A2 Ag	Clonidine	0.1	2	2a, 3b, 4b, 5b, 6b, 7a
	b	25	62	8	8			0.2	2	
	c	25	62	8	8			0.4	2	
Schlegel 1989*^24^		40	60	5	5	A2 Ag	Guanfacine	0.5–1	2	2a, 3a, 4b, 5c, 6c, 7a
Huff 1996^37^	a	Unknown	Unknown	92	91	A2 Ant	Besipirdine	10	12	1b, 8b
	b	Unknown	Unknown	92	91			40	12	
Rinne 2017^38^	a	59	72	33	34	A2 Ant	ORM-12741	30–60	12	2b, 4c, 5d, 6d, 7c
	b	59	72	33	34			100–200	12	
Peskind 2005^75^		80.6	85	17	14	B Ant	Propranolol	120	6	9b, 8a, 10a
Mean/total over all studies		56.7	75.8	45.9	45.1	N/A	N/A	N/A	12.9	N/A

Drug: NRI=norepinephrine reuptake inhibitor; A1 Ant=alpha1 adrenergic receptor antagonist; A2 Ag= alpha2 adrenergic receptor agonist; B Ant=Beta adrenergic receptor antagonist/blocker; A2 Ant=alpha2 adrenergic receptor antagonist. Outcomes: Global cognition: 1a=Mini-Mental State Examination; 1b=Alzheimer’s Disease Assessment Scale—Cognitive Subscale. Attention: 2a=Digit Span Forwards; 2b=Continuity of Attention; 2c=Trails A. Visuospatial: 3a=Benton Visual Retention—No. Correct; 3b=Visual Retention Test—Delayed Recall; 3c=15 Objects Test; 3d=Spatial Recognition Memory (latency). Semantic Memory: 4a=Action Verbal Fluency Test; 4b=Supermarket fluency; 4c=Category fluency test. Episodic Visual Memory: 5a=Benton Visual Retention; 5b=Visual Retention Test—Delayed Recall; 5c=Quality of Episodic Memory. Episodic Verbal Memory: 6a=Hopkins Verbal Learning Test—Revised Delayed Recall; 6b=Verbal Learning Delayed Recall; 6c=Rey Verbal Learning—Delayed Recall; 6d=Controlled Oral Word Association Test; 6e=Wechsler Memory Scale—Logical Memory Delayed Recall. Executive Functions and Working Memory: 7a=Digit Span Backwards; 7b=Wechsler Paired Associates; 7c=Quality of Working Memory; 7d=Trails B.

General behaviour/neuropsychiatric symptoms: 8a=The Neuropsychiatry Inventory—Total; 8b=Alzheimer’s Disease Assessment Scale—Non-Cognitive Subscale. Apathy: 9a=Apathy Evaluation Scale; 9b=The Neuropsychiatry Inventory—Apathy; 9c=The Frontal Systems Behaviour Scale—Apathy. Agitation: 10a=The Neuropsychiatry Inventory—Agitation; 10b=The Cohen-Mansfield Agitation Inventory.

*Cross-over design.

†The same trial reported across different publications.

‡Mild cognitive impairment due to Alzheimer’s disease.

**Table 2:** Inclusion and exclusion criteria and search strategy* for studies investigating the use of noradrenergic treatments in neurodegenerative conditions

Inclusion criteria	Exclusion criteria
Study published between 1980 and 22 December 2021	Study published prior to 1980
Peer reviewed	Editorials, review articles, letters, or case reports
Prospective trial	Conference abstracts
Placebo controlled	No placebo group
n>1	Single dose studies
Any age	Not in English
Study includes predominantly patients with the included diagnoses (or subgroup analysis including patients with the included diagnoses)	Poorly defined patient cohort for example, ‘dementia’
Study reports either a change in recognised score of cognition and/or psychological/psychiatric symptoms/behaviour	Data not accessible, including after request from authors if necessary
For studies reporting duplicated data, the most recent or most comprehensive publication to be indexed	Duplicate data
English language	
Study of drug with principally noradrenergic action	

*Search was done using the following terms ($is used as a truncation command): ((Alzheimer$ or Parkinson$ or “Lewy bod$” or “Frontotemporal d$” or “progressive supranuclear palsy” or “mild cognitive impairment”) and (cogniti$ or behav$ or psychiatric or psychological or memory or attention) and (noradren$ or norepineph$ or epineph$ or adrenergic or “vesicular monoamine transporter inhibitor” or catechol-O-methyltransferase or “Phenylalanine hydroxylase inhibitor” or “Tyrosine hydroxylase inhibitor” or "Aromatic L-amino acid decarboxylase inhibitor” or “Dopamine-beta-hydroxylase inhibitor” or “Phenylethanolamine N-methyltransferase” or guanfacine or atomoxetine or methylphenidate or clonidine or yohimbine or prazosin or mirtazapine) and (trial or control$ or experimental or placebo).mp.)

#### Patient populations

The six conditions were chosen as neurodegenerative diseases with significant LC degeneration.^3 29^ Given the involvement of the LC in early onset Alzheimer’s disease, we elected to have no lower age limit.^30^

#### Trial designs

We focused on prospective clinical trials of the effect of a chronic course of medication, excluding single-dose studies. While single-dose experimental studies are valuable in understanding the mechanisms of actions of drugs, particularly when performed with ancillary neurophysiology or neuroimaging, they are not informative about clinical efficacy of chronic treatment.

#### Drugs

**Figure 2:**
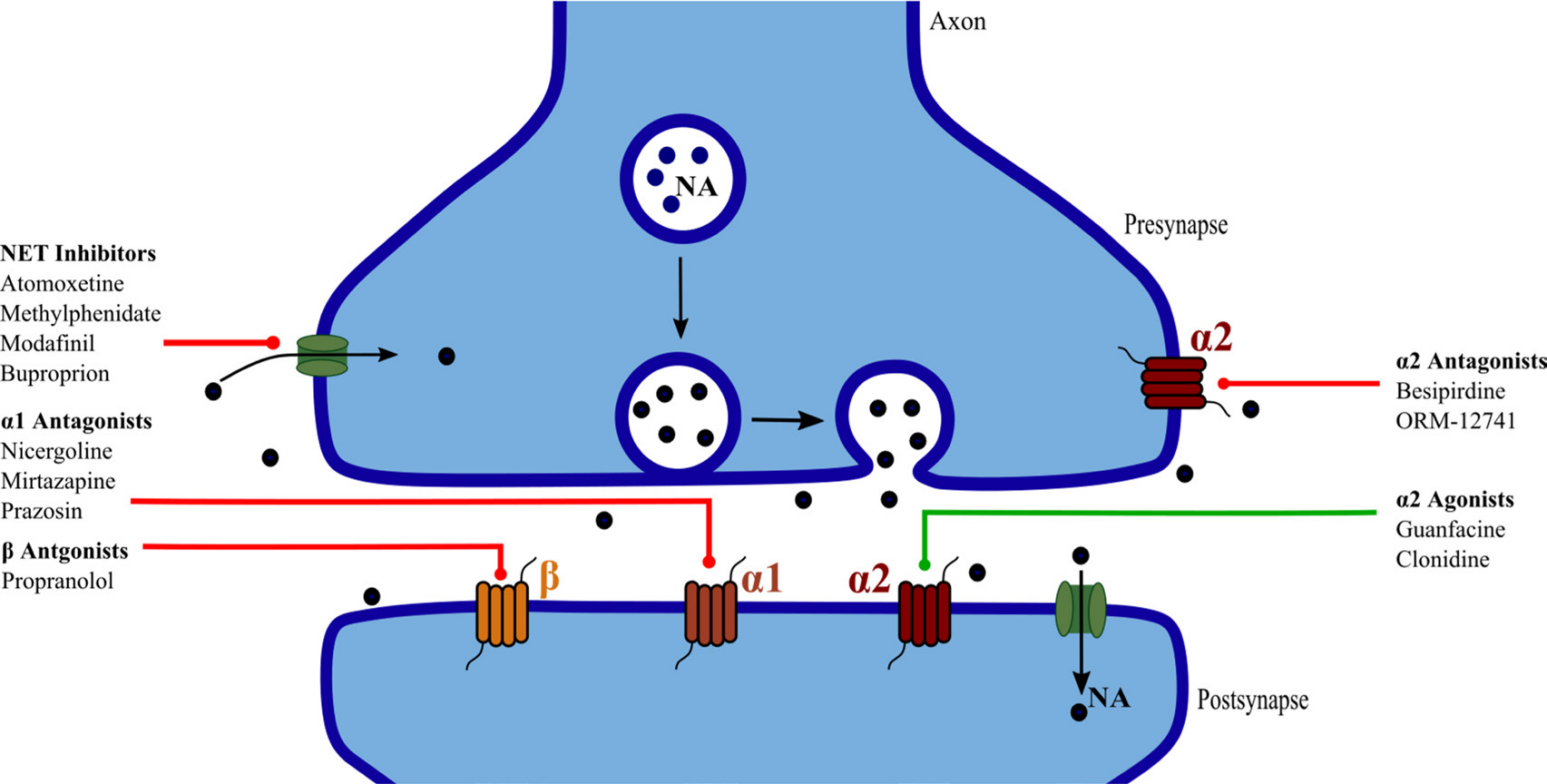
Schematic showing release of norepinephrine (NA) across the synapse, action at the three receptor subtypes and reuptake through the norepinephrine transporter (NET). Presumed site of therapeutic action of the drugs included in this review are shown.

Drugs with activity across multiple neurotransmitter systems were not included unless there was evidence of predominant noradrenergic action (figure 2). Examples of drugs excluded for this reason are olanzapine and trazodone; while they have slight noradrenergic actions, these are of secondary significance compared with dopaminergic and serotonergic effects.^31 32^ Monoamine oxidase type-A inhibitors were included, whereas type-B inhibitors were not, given the former’s relative selectiveness for NA.^33^ The noradrenergic system’s involvement in L-3,4-dihydroxyphenylalanine’s effects is not strong, and we excluded it.^29^ While methylphenidate is partly dopaminergic, it has a significant inhibitory effect on NA reuptake^21^ and hence was included. Mirtazapine was included.^34^

#### Outcome measures

Cognitive outcomes included measures of ‘global cognition’ on screening tests and specific cognitive domains (attention, episodic verbal memory, episodic visual memory, executive functions and working memory, semantic memory and visuospatial abilities). Global measures of behaviour and neuropsychiatric symptoms; agitation and apathy, were included (table 1). We included objective-observed measures, including carer-based assessments, but not self-rated outcomes in which patients are asked to report their symptoms, for example, of depression or anxiety.

### Search strategy

We searched MEDLINE and Embase from 1980 to 22 December 2021 using a combination of controlled vocabulary (eg, Medical Subject Headings) and free-text terms. We manually searched further information sources, including published studies or reviews, conference abstracts and supplementary notes. We searched ClinicalTrials.gov to identify trials with published results.

### Study selection and data extraction

Four reviewers (MCBD, MdG, BG and IO) independently screened titles and abstracts. Potentially eligible studies were then discussed between the reviewers before deciding on inclusion. Arbitration was conducted by RH and PAM. Data were extracted from full texts by two reviewers (MCBD and MdG) and additional data were requested from authors where required.

Outcome measures for each domain were selected based on a hierarchy determined by their frequency of use across included studies. For example, for the global cognition outcome measure, Mini-Mental State Examination (MMSE) was used in seven Alzheimer’s disease studies and therefore included in the analysis for these studies. Two Alzheimer’s disease studies reporting measures of global cognition employed the Alzheimer’s Disease Assessment Scale—Cognitive Subscale (ADAS-Cog), and so the results from this were included in analysis. For apathy, four Alzheimer’s disease studies used the Apathy Evaluation Scale (AES), and this was extracted where available. The next most common index of apathy was the Neuropsychiatry Inventory—Apathy (NPI-A), while one study used the Frontal Systems Behaviour Scale—Apathy (FrSBe-A). For selection of outcome measures for the cognitive subdomains the most frequently used measures were prioritised, after which, those measures deemed most similar were chosen from the remaining studies. See table 1 for details on the included outcome measures.

Drugs were grouped by principal mechanism of action (table 1). Analyses were run for all studies investigating specific classes of drugs, where there were enough (>2) to do so (see online supplemental material).

### Study quality assessment

Two reviewers (BG and IO) assessed the quality of the studies using the National Heart, Lung, and Blood Institute Quality Assessment of Systematic Reviews and Meta Analyses.^35^ This comprises 14 questions on blinding, randomisation, equality between study arms, drop-out rates, outcome measures, power and analyses. Raters score each question as 1 if the methodology is suboptimal, and rank studies as ‘good’, ‘fair’ or ‘poor’ if they scored 0, 1 or >1 respectively across the 14 questions.

### Statistical analysis

To evaluate treatment efficacy, our meta-analysis used Review Manager V.5.4.^36^ For each outcome measure, we calculated the change in group means for drug and placebo groups in each study, from baseline to the final timepoint. Where the relevant information was specified in the studies, we chose to use datasets that had excluded the participants who dropped out before the final time point. For outcome measures in which a negative change in score indicated an improvement, change scores were multiplied by −1; and are shown as such in the figures. As all outcomes were continuous, we present the calculated standardised mean differences (SMDs) with 95% CIs using an inverse variance random effects model. If a study used multiple intervention groups (different doses), we treated each study arm as a separate trial and compared them against the same control group.^23 37 38^ Where SDs for the change in mean were not reported, SDs were imputed using formulas for continuous outcomes in the Cochrane Handbook.^39^ SMDs were used because studies used different measures for the same outcome, except for digit span forwards and backwards where the mean difference was calculated. On the assumption that clinical and methodological heterogeneity was present and could influence outcomes, we used random effects meta-analysis models to estimate SMDs.

Heterogeneity was measured using the I*^2^* statistic. Funnel plots were created using JASP^40^ to graphically represent effect sizes and identify asymmetry resulting from publication bias. This was quantified using Egger’s tests, with the caveat that tests for asymmetry with <10 studies are likely to be underpowered.^41^ Studies that differed significantly from the pooled effect in that their 95% CIs did not overlap with the CIs of the pooled effect, were considered outliers. The effect of their exclusion on the pooled effect size and study heterogeneity was explored.

Post hoc meta-regression analyses were conducted to define whether age, gender, duration of treatment and year of publication had any effect on the results obtained for global cognition and apathy. Meta-regressions were conducted for each covariate separately. For each meta-regression, the number of studies included in the model, the covariate estimate (β), the p value and the proportion of variance explained (R^2^) were reported(online supplemental tables S4 and S5).

## Results

### Study characteristics

Table 1 shows the baseline characteristics of included trials. We focus on the 19 trials of Alzheimer’s disease and MCI, as the search revealed only four eligible PD trials, one in FTD, and none in Lewy Body dementia or Progressive Supranuclear Palsy. Details of the non-Alzheimer’s studies are shown in online supplemental table S1.

The Alzheimer’s disease studies were prospective randomised controlled trials, with treatment duration between two and 52 weeks. Study participant number ranged from 5 to 346 and the mean participant age ranged from 60 to 85 years. The most common drugs were norepinephrine reuptake inhibitors (NRIs; nine studies), followed by α_1_-AR antagonists (four studies), α_2_-AR agonists (three studies), α_2_-AR antagonists (two studies) and β-AR antagonists/blockers (one study).

### Study quality assessment

Six studies were of ‘good’ quality, with seven ‘fair’ and six ‘poor’ (online supplemental table S2); all were included in the analysis. Methods of randomisation were adequate in all but one study (unable to determine in five). Four studies had a high drop-out rate (>20%) from the treatment arm and were considered ‘poor’ overall. Only five included studies reported a sufficiently large sample size to detect a significant difference in primary outcome measure with 80% power; four of these were considered ‘good’ overall, with one ‘fair’ (the remaining two ‘good’ papers did not report on their power).

### Outcome measures

#### Global cognition

**Figure 3:**
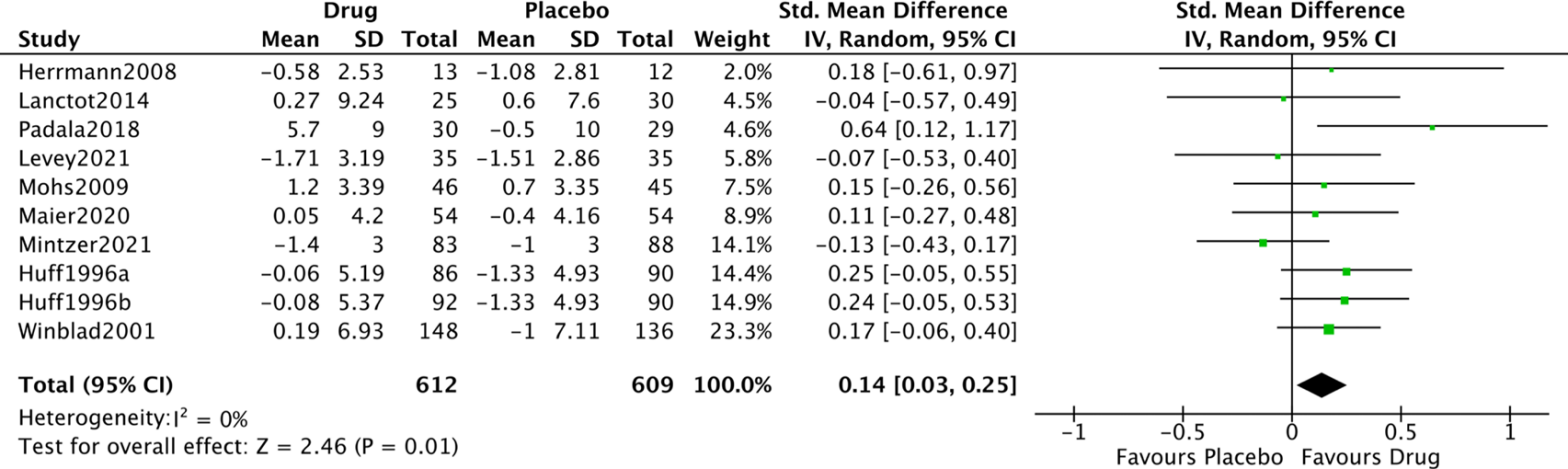
Forest plot of noradrenergic drugs on global cognition. Comparison of drug and placebo for effect on global measures of cognition between baseline and end of treatment. IV, inverse variance.

Ten studies assessed the change in global cognition from baseline in Alzheimer’s disease (figure 3). The overall pooled effect size showed a small^42^ but significant positive effect of noradrenergic drugs compared with placebo (SMD: 0.14, 95% CI: 0.03 to 0.25, p=0.01; I^2^=0%). After removal of the single ‘poor’ quality study, the effect size remained unchanged (SMD: 0.14, 95% CI: 0.02 to 0.27, p=0.03; I^2^=11%). For context, this effect size sits between that of cholinesterase inhibitors in Alzheimer’s disease (SMD: 0.38, 95% CI: 0.28 to 41.1; I^2^=41.1%),^43^ and MCI (SMD: 0.06, 95% CI: −0.08 to 0.20; I^2^=76%).^44^

#### Cognitive subdomains

**Figure 4:**
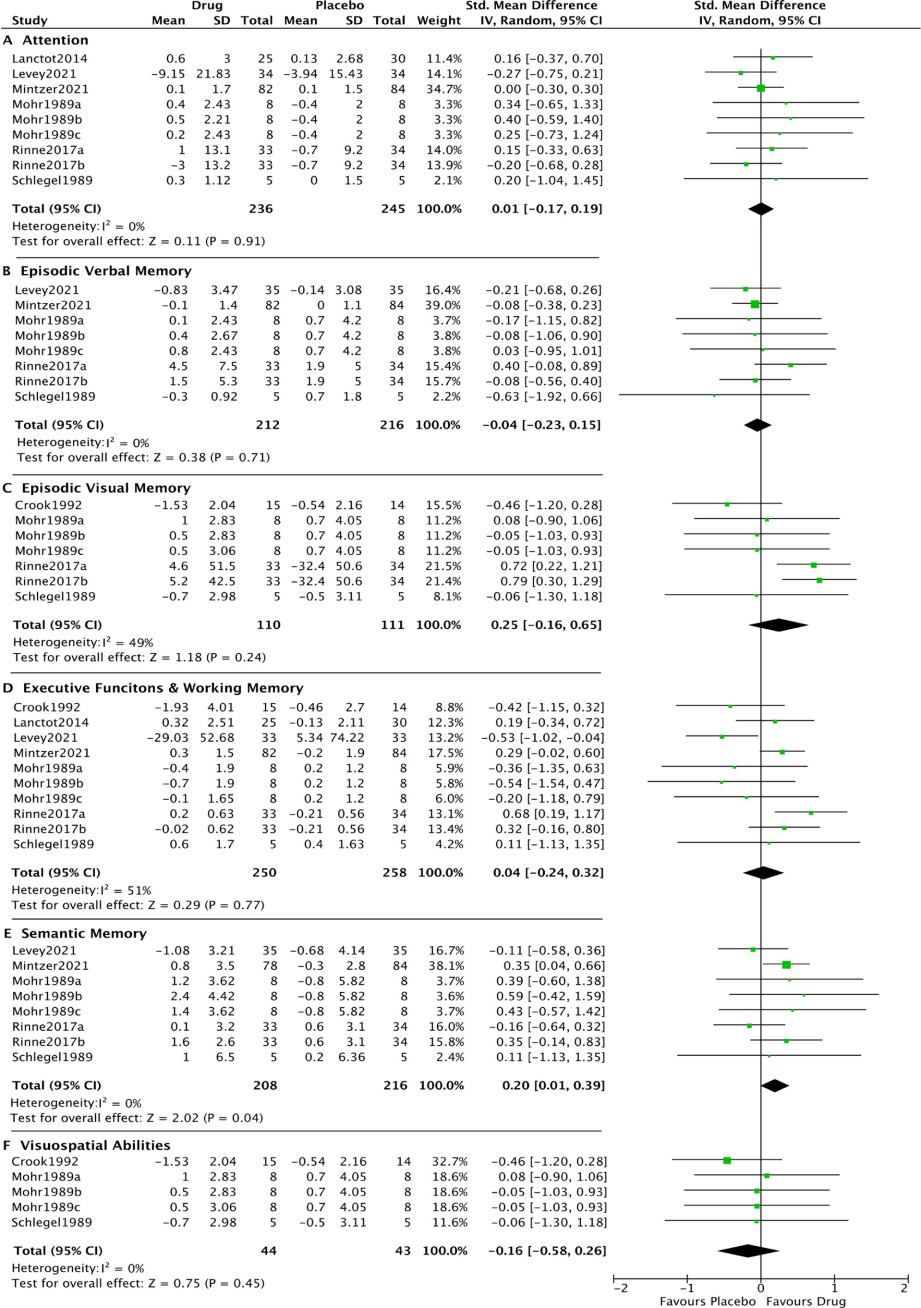
Forest plot of noradrenergic drugs on cognition subdomains. Comparison of drug and placebo for effect on cognitive subdomains between baseline and end of treatment. IV, inverse variance.

There was a significant, medium-sized^42^ positive effect of noradrenergic drugs on semantic memory (SMD: 0.20, 95% CI: 0.01 to 0.39, p=0.04; I^2^=0%). After removal of the ‘poor’ quality studies, the result across the remaining four studies was not significant (SMD: 0.14, 95% CI: −0.13 to 0.41, p=0.32; I^2^=38%). The overall pooled effect was not significant for measures of attention (SMD: 0.01, 95% CI: −0.17 to 0.19, p=0.91; I^2^=0%), episodic verbal memory (SMD: −0.04, 95% CI:−0.23 to 0.15, p=0.71; I^2^=0%), episodic visual memory (SMD: 0.25, 95% CI:−0.16 to 0.65, p=0.24; I^2^=49%), executive functions and working memory (SMD: 0.04, 95% CI:−0.24 to 0.32, p=0.77; I^2^=51%) and visuospatial abilities (SMD: −0.16, 95% CI:−0.58 to 0.26, p=0.45; I^2^=0%) (figure 4).

**Figure 5:**
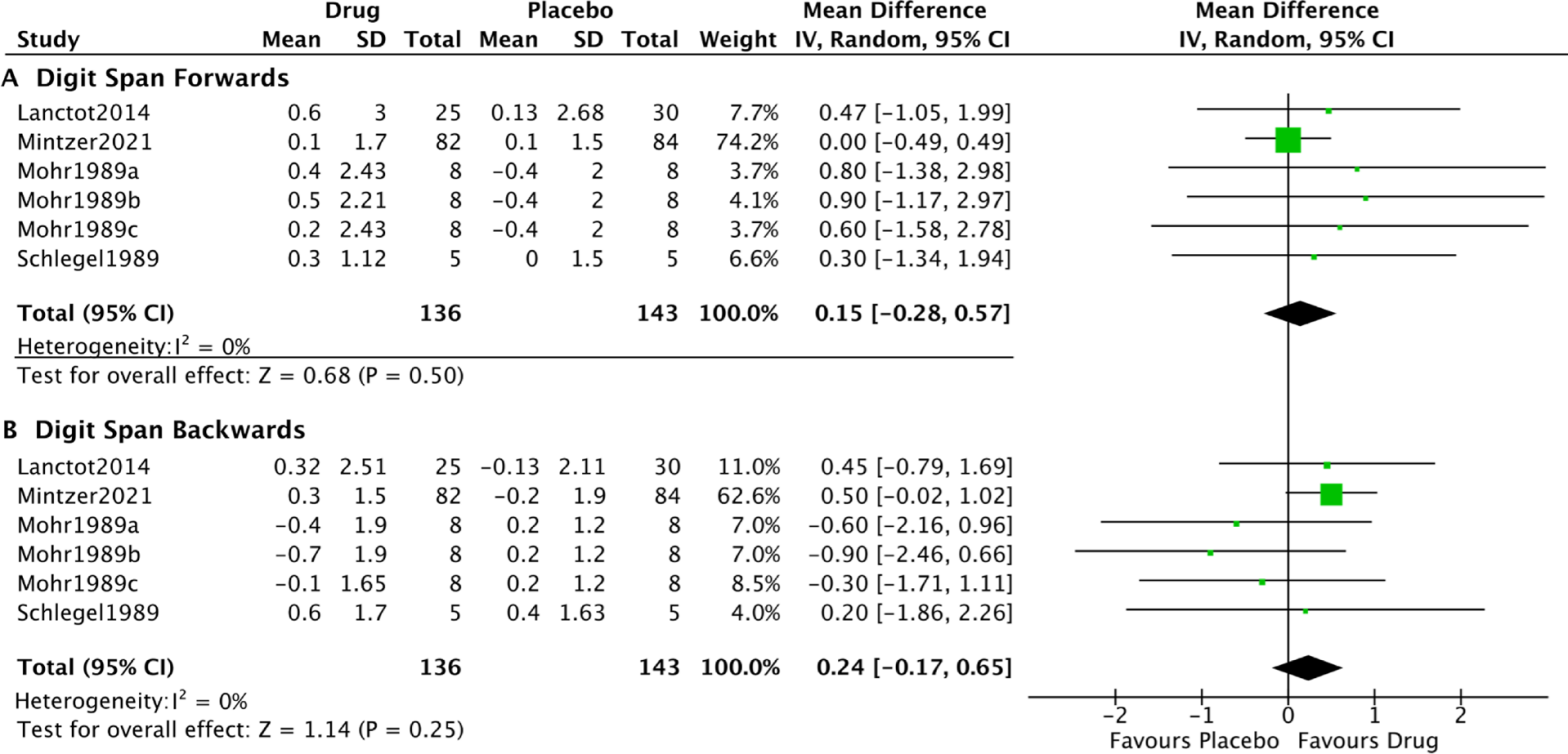
Forest plot of noradrenergic drugs on digit span. Comparison of drug and placebo for effect on global measures of cognition between baseline and end of treatment. IV, inverse variance.

Subanalyses assessed the noradrenergic treatment effect on digit span forwards and digit span backwards tasks, as putative measures of attention and working memory respectively. The pooled effect was not significant (digit span forwards: SMD: 0.15, 95% CI: −0.28 to 0.57, p=0.50; I^2^=0%. Digit span backwards: SMD: 0.24, 95% CI: −0.17 to 0.65, p=0.25; I^2^=0%) (figure 5).

Inspection of the funnel plots for global cognition and the sub-domains did not identify asymmetry to a degree that suggests publication bias, except for episodic visual memory. Egger’s tests were not significant (p>0.05), except for episodic visual memory (p=0.01, z=−2.582). No outliers were identified for the analyses of global cognition or the subdomains.

#### Neuropsychiatric symptoms and apathy

**Figure 6:**
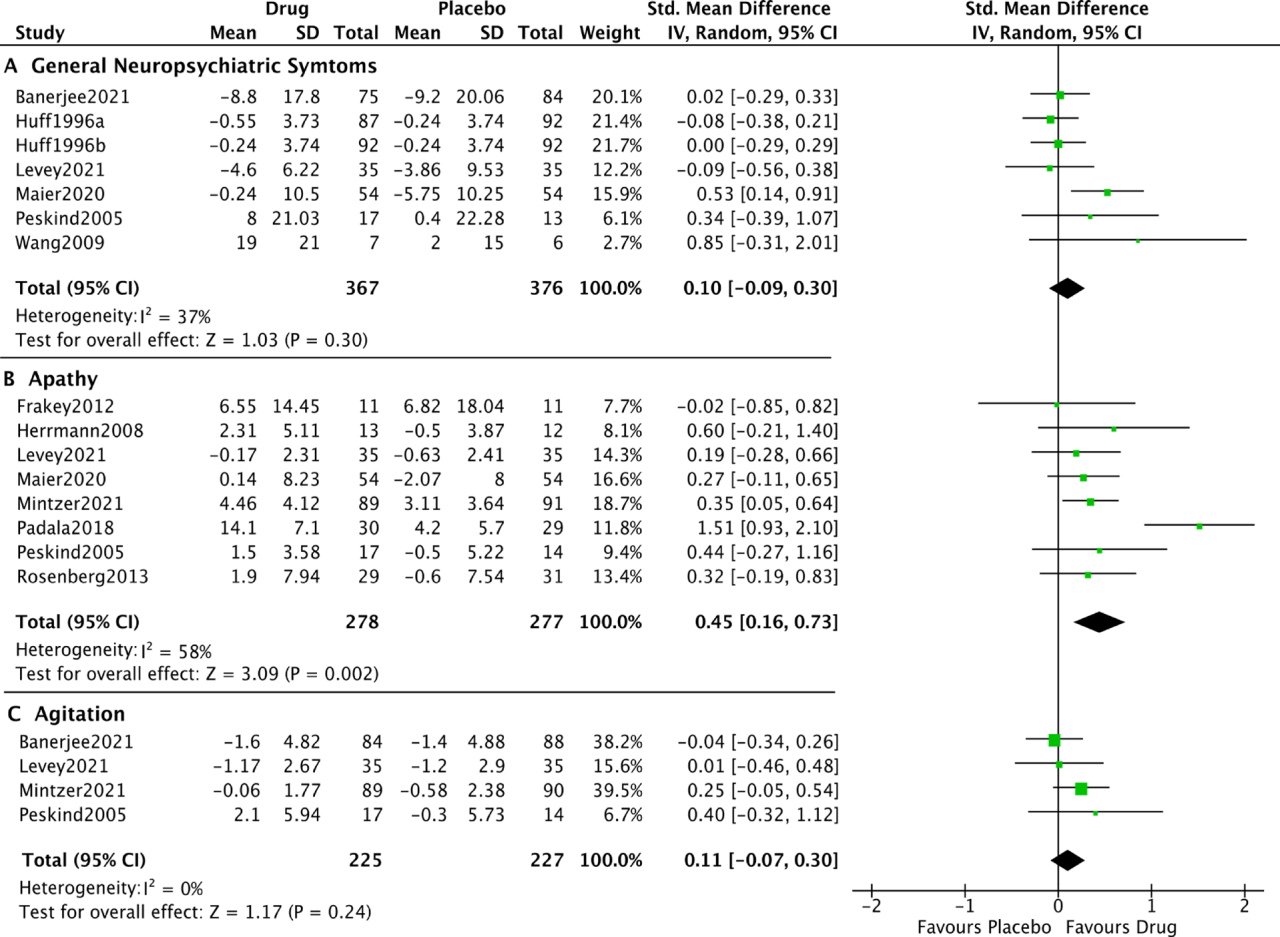
Forest plot of noradrenergic drugs on neuropsychiatric symptoms. Comparison of drug and placebo for effect on global measures of cognition between baseline and end of treatment. IV, inverse variance.

Analyses of neuropsychiatric symptoms are summarised in figure 6. The apathy meta-analysis included eight trials and detected a large^42^ positive effect of noradrenergic drugs (SMD: 0.45, 95% CI: 0.16 to 0.73, p=0.002; I^2^=58%), although results were limited by potential heterogeneity. The effect size of one study^45^ was identified as an outlier and its exclusion from the analysis reduced the I*^2^* heterogeneity from 58% (p=0.02) to 0% (not significant, p=0.96), and the SMD to 0.31 (95% CI: 0.13 to 0.48, p<0.001). After removal of the two ‘poor’ quality studies, the effect size increased slightly (SMD: 0.49, 95% CI: 0.10 to 0.88, p=0.01; I^2^=69%).

The pooled effect provided no support for an effect of noradrenergic drugs on agitation (SMD: 0.11, 95% CI: −0.07 to 0.30, p=0.24; I^2^=0%) or general measures of neuropsychiatric symptoms (SMD: 0.10, 95% CI: −0.09 to 0.30, p=0.30; I^2^=37%) compared with placebo. Egger’s tests were not significant (all p>0.05).

Meta-analyses of pooled studies across diagnostic groups, subgroup analysis for global cognition in PD and subgroup analysis of individual drug classes are reported in the online supplemental material.

### Meta-regression

Meta-regression analyses showed that none of mean age, sex, duration of treatment and year of publication were significantly associated with effect size differences in either the global cognition or apathy meta-analyses (p>0.05) (online supplemental tables S4 and S5 and figure S6). Results for global cognition were limited by insufficient power and one study could not be included as age and sex were not reported,^37^ as was one study for the investigation of the influence of gender on the apathy results.^46^

## Discussion

This systematic review and meta-analysis considered noradrenergic pharmacotherapies in Alzheimer’s disease, for improving cognition, behaviour and neuropsychiatric symptoms. We found moderate quality evidence from 1300 patients that noradrenergic drugs improve global cognition as measured by the MMSE or ADAS-Cog. Although no effect was seen on overall measures of neuropsychiatric symptoms, a significant improvement in apathy was seen, as measured by the AES, NPI-A or FrSBe-A. Although the apathy analysis only included 425 patients, it was particularly robust, and evidence of benefit remained after outlier removal. Apathy is a common and prognostically adverse feature of Alzheimer’s disease.^13^ Methylphenidate was the most frequently trialled medication for apathy. Its actions are likely to be mediated by prefrontal-striato-thalamo-cortical circuits.^47^ While there may be a dopaminergic component to this effect, methylphenidate leads to a proportionately greater increase in NA than dopamine in the rat prefrontal cortex.^48^ Preclinical and clinical experimental evidence indicates complementary roles for dopamine and NA in controlling motivation and decisions. Indeed, the relationship between neuronal activity and effort is more pronounced in the noradrenergic LC than dopaminergic substantia nigra^49^; while NRIs moderate apathy in PD in proportion to LC integrity.^50^ There is a dynamic, likely bidirectional, relationship between apathy and cognitive impairment. In Alzheimer’s disease, cognitive impairment can result in reduced motivation and vice versa,^51^ although in other disorders like FTD, apathy is more predictive of cognitive decline than vice versa.^52^ Single study evidence that noradrenergic treatments improve both domains is inconsistent.^13 45^ Our meta-analysis suggests that modulating the LC-NA system can improve both cognition and apathy.

With regards to agitation, the results do not provide evidence for a significant effect of noradrenergic medication. This may be related to the inclusion of trials where agitation was not a prominent symptom in the trial population as we included data that addressed any of our stated outcome measures, even if they were not the primary outcome of the respective study. This may have affected the results of the agitation meta-analysis, where only two of the four included studies were primarily investigating agitation. Although this may not have qualitatively affected the effect size estimate for agitation, this increased study heterogeneity. There is scientific rationale to support the potential for noradrenergic therapies targeting this symptom—whether it be enhancement or suppression of the system.^53^ Therefore, further targeted studies are warranted, with the need for clarification of outcome measures, greater power and standardised symptom classification.^53^

### Interpretation and implications

This meta-analysis suggests that drug repurposing with established noradrenergic treatments, such as atomoxetine, methylphenidate and guanfacine, may benefit people with Alzheimer’s disease, particularly given existing evidence of their relative safety in clinical practice, and pharmacological target engagement.^54^ However, several factors may explain the variability in results across studies. These factors are important to bear in mind when designing future clinical trials.

First, cognitive performance is proposed to be optimal at an intermediate level of noradrenergic tone, in accordance with the Yerkes–Dodson arousal curve.^55^ Therefore, it is likely that noradrenergic treatments are maximally effective only at a specific dose; or only in patients with a sufficient degree of LC-NA dysfunction so as to not to induce a state of over-activity of the LC-NA system. Such overactivity might lead to agitation and anxiety, or worsening of cognition.^27 56^ For example, atomoxetine does not merely produce a general increase in synaptic NA levels,^54^ but increases the likelihood that patients will be at an ‘engaged’, intermediate state of noradrenergic tone. In promoting this state, LC neurons are rendered less tonically active with heightened neural gain. This can increase responsiveness to stimuli, increase network integration, and improve cognitive performance.^57^ However, in clinical psychopharmacological studies, there is baseline-dependency such that the effect of atomoxetine on connectivity and cognition depends on severity of disease and integrity of the LC. Such baseline-dependency may apply in Alzheimer’s disease too, calling for stratification tools in clinical trials and practice.^27 58^

Second, we analysed the effects of both AR agonists and antagonists. While it might seem counterintuitive, there is evidence for effectiveness of different treatment strategies. Recall that some pre-synaptic receptors are inhibitory on NA release, such that antagonism can paradoxically increase NA neurotransmission. In addition, receptor subtypes can have opposing actions in different locations within the brain. With the inverted-U shape response to noradrenergic stimulation, stimulation and inhibition may be helpful at different stages of illness and for different cognitive or behavioural domains. Further evidence is required to differentiate the effects of individual drugs. At present, neither our meta-analysis of global cognition nor apathy, both of which showed positive effects of treatment, included trials evaluating agents that had opposing effects on the same receptor subtype.

Third, optimal symptomatic treatment of AD may not come from targeting a single neurotransmitter system. Yu and Dayan’s theoretical account of synergistic cholinergic and noradrenergic systems in attentional processes suggests that noradrenergic therapy may prove most effective when used in tandem with cholinergic approaches.^59^ This approach was taken with the trial by Mohs and colleagues included here.^22^ There are ongoing studies looking at combination therapies with noradrenergic and cholinergic agents based on this principle, including a Phase 3 trial of guanfacine as an 'add-on' to cholinergic treatment (NCT03116126).^60^ A number of included studies involved drugs with a combination of noradrenergic and dopaminergic action, such as methylphenidate, and it is possible that dual stimulation leads to greater clinical efficacy, particularly for apathy, than more targeted approaches. Direct comparison between selective noradrenergic and dopaminergic agents and a less targeted treatment such as methylphenidate is required to clarify this.

Fourth, it is important to consider the potential benefits of noradrenergic treatments beyond cognitive and behavioural effects. Several studies have shown that with dysfunction in the LC-NA system in Alzheimer’s disease, there is a loss of endogenous anti-inflammatory effects and of the capacity for amyloid beta peptide (Aβ) degradation and clearance^1 61 62^ that is normally promoted by NA. Noradrenergic deficits may thereby lead to an increase in Aβ production. In the context of reduced NA, the toxic inflammatory effect of Aβ and tau are heightened, exacerbating neurodegeneration.^63 64^ NA also regulates microglial surveillance and synaptotoxicity,^6^ where microglia activation is predictive of faster cognitive decline of people with Alzheimer’s disease and MCI.^65^ Therefore, noradrenergic treatments might have both symptomatic and disease-modifying effects. It is important to note that these effects can be positive or negative. Brain activity leading to the release of NA can lead to the aggregation of Aβ and tau, and thus there is potential for both deleterious and disease-modifying effects.^62^ Future trials aiming to measure effects on pathological progression in addition to symptomatic improvement will need sufficient treatment duration to detect any such effect.

Fifth, the benefits of noradrenergic treatment need to be weighed against potential adverse effects, including cardiac risks, especially in people with multimorbidities. Noradrenergic reuptake inhibition and agonism have the potential to increase heart rate, blood pressure and cardiac risk. Even in older adults, the actual changes in rate and pressure seem minimal where reported in trials, but screening and risk-stratification may be required.

### Strengths and limitations

The strengths of our study include the following: the comprehensive approach to the search that discovered 19 Alzheimer’s disease studies, including 1811 patients, that investigated the effect of noradrenergic drugs on cognition and behaviour; pre-planned subgroup analyses to explore the effect of treatments on a range of outcome measures; and the use of established methods to assess effect of outliers and quality of evidence.

There are nonetheless limitations to our study. The inclusion criteria used a threshold of drugs’ noradrenergic action, which was not based on fixed pharmacokinetic or pharmacodynamic metrics. Rather, we considered the relative action of drugs on noradrenergic and non-noradrenergic systems at the doses used in current licenced applications of the drugs. We acknowledge that other drugs have some noradrenergic activity, even if not enough to be included here. The treatments included medications that have varying mechanisms within the noradrenergic system, including both AR agonists and antagonists, as well as some non-noradrenergic activity. It is uncommon for drugs used in clinical practice or human trials to have exact specificity of pharmacological action. Critically for the conclusions drawn here, the commonality of effect of all the compounds included here lies in their shared noradrenergic action. Different measures (for the same outcome) were used across studies. The change in mean score for each outcome measure was reported at the group level in most studies, whereas a change at the individual level may have yielded more accurate results and allowed estimation of state-dependency in relation to disease severity. We did not model baseline performance or symptom severity, which may account for some of the variation in treatment response. Regarding study quality, only 6 of the 19 being rated as ‘good’. We propose that further phase 3 clinical trials are warranted in Alzheimer’s disease, with optimal design and outcomes, with consideration of pragmatic baseline stratification with a view to regulatory benchmarking. In view of the impact of non-Alzheimer dementias on the LC-NA system, clinical trials of other diagnostic groups are also indicated, with a strong rationale for expected treatment potential.

## Conclusions

In patients with dementia or MCI caused by Alzheimer’s disease, pharmacotherapies targeting the noradrenergic system can improve cognition and apathy. These therapies do not appear to have any beneficial effects on attention or episodic memory. Based on this meta-analysis, and recognition of the importance of LC-NA system in multiple neurodegenerative diseases, there is a case for further clinical trials of noradrenergic agents in Alzheimer’s disease and other neurodegenerative conditions.

## Data Availability

Data are available upon reasonable request.
