# Supplementary material for: Cognitive and neuropsychiatric effects of noradrenergic treatment in Alzheimer’s disease: systematic review and meta-analysis
Source: J Neurol Neurosurg Psychiatry. 2022 Jul 5;93(10):1080–90. doi: 10.1136/jnnp-2022-329136 (PMC9484390; doi:10.1136/jnnp-2022-329136)
Supplement: Supplementary file 1 [file jnnp-2022-329136supp001.pdf]

Supplementary

Table S1. Characteristics of non-Alzheimer's disease studies

Study	Participants					Intervention				Outcomes included
				N						
	Diagnosis	% Female	Mean age, years	Drug	Placebo	Class	Drug	Daily dosage, mg	Duration, weeks	
Weintraub2010[1]	PD	34	64.3	28	27	NRI	Atomoxetine	80	8	1a, 9e
Moreau2012*[2]	PD	79.7	63.5	35	34	NRI	Methylphenidate	1/kg	12	1c, 2c, 9d
Bédard1998†[3]	PD	Unknown	68.6	9	9	A1 Ag	Naphtoxazine	25	1	2d, 3e, 4d, 7d
Svenningsson2020[4]	PD	12.5	72	25	7	A2 Ant	IRL752	750-900	4	1c, 7e
Fremont2020†[5]	FTD	50	63.1	14	14	COMT I	Tolcapone	100-200	1.1	1d, 8a, 9b, 10a

PD = Parkinson's disease, FTD = Frontotemporal dementia.
NRI = Noradrenaline reuptake inhibitor, A2 Ant = alpha2 adrenergic receptor antagonist, A1 Ag = alpha1 adrenergic receptor agonist, COMT I = Catechol-O-methyltransferase inhibitor.
Outcomes: *Global cognition:* 1a = Mini-Mental State Examination; 1c = The Movement Disorder Society-Sponsored Revision of the Unified Parkinson's Disease Rating Scale – Section 1; 1d = Repeatable Battery for the Assessment of Neuropsychological Status. *Attention:* 2c = Simple Reaction Time; 2d = Continuous Performance Task – Reaction Time. *Visuospatial:* 3e = 15 Objects Test. *Language:* 4d = Verbal Fluency. *Executive Functions and Working Memory:* 7d = Stroop Test; 7e = Cambridge Neuropsychological Test Automated Battery – Total Errors.
General Behaviour/Neuropsychiatric Symptoms: 8a = The Neuropsychiatry Inventory – Total. *Apathy:* 9b = The Neuropsychiatry Inventory – Apathy; 9d = Lille Apathy Rating Scale; 9e = Apathy Scale. *Agitation:* 10a = The Neuropsychiatry Inventory – Agitation.
†Crossover design. For Fremont2020, all patients took a course of placebo followed by a course of drug, or vice versa. The change in outcome measure was taken as the end of each treatment period minus the score at the pre-study baseline
*Reported medians and these were converted to means as per the Cochrane Handbook[6 7].

Table S2. Quality assessment using the NIH Quality Assessment Tool for Controlled Intervention Studies

Study	Patient group	Quality assessment question														Quality rating
		1	2	3	4	5	6	7	8	9	10	11	12	13	14	
Amaducci1999	AD	Y	CD	Y	Y	Y	Y	Y	Y	Y	Y	Y	NR	Y	Y	Good
Banerjee2021	AD	Y	Y	Y	Y	Y	Y	N	Y	Y	Y	Y	N	Y	Y	Poor
Crook1992	AD	Y	NR	Y	Y	Y	Y	Y	Y	Y	Y	Y	NR	Y	Y	Good
Frakey2012	AD	Y	Y	Y	Y	Y	Y	Y	Y	Y	Y	Y	N	Y	Y	Fair
Herrmann2008	AD	Y	NR	Y	Y	Y	NR	Y	Y	Y	Y	Y	N	Y	Y	Fair
Huff1996	AD	Y	NR	Y	Y	Y	Y	Y	Y	Y	Y	Y	Y	Y	Y	Good
Lanctot2014	AD	Y	Y	Y	Y	Y	Y	Y	Y	Y	Y	Y	Y	Y	Y	Good
Levey2021	MCI (AD)	Y	Y	Y	Y	Y	Y	Y	Y	Y	CD	Y	N	Y	NA	Fair
Maier2020	AD	Y	Y	Y	Y	Y	Y	N	Y	Y	Y	Y	N	Y	Y	Poor
Mintzer2021	AD	Y	Y	Y	Y	Y	Y	Y	Y	Y	Y	Y	Y	Y	Y	Good
Mohr1989	AD	N	N	Y	Y	Y	NA	Y	NA	Y	Y	CD	N	NA	NA	Poor
Mohs2009	AD	Y	Y	Y	Y	Y	Y	Y	Y	Y	Y	Y	N	Y	Y	Fair
Padala2018	AD	Y	Y	Y	Y	Y	Y	Y	Y	Y	Y	Y	N	Y	Y	Fair
Peskind2005	AD	Y	Y	Y	Y	Y	Y	N	N	Y	Y	Y	N	Y	Y	Poor
Rinne2017	AD	Y	Y	Y	Y	Y	Y	Y	Y	Y	Y	Y	N	Y	Y	Fair
Rosenberg2013	AD	Y	Y	Y	Y	Y	Y	Y	Y	Y	Y	Y	Y	Y	Y	Good
Schlegel1989	AD	N	CD	Y	Y	Y	NA	NA	NA	Y	Y	Y	N	Y	NA	Poor
Wang2009	AD	Y	Y	Y	Y	Y	Y	N	Y	Y	Y	Y	N	Y	Y	Poor
Winblad2001	AD	Y	Y	Y	Y	Y	Y	Y	Y	Y	N	Y	Y	NR	Y	Fair
Weintraub2010	PD	Y	Y	Y	Y	Y	Y	N	N	Y	N	Y	N	CD	Y	Poor
Svenningsson2020	PD	Y	Y	Y	Y	Y	Y	Y	Y	Y	Y	Y	N	Y	Y	Fair
Bedard1998	PD	N	NA	Y	NA	NA	Y	NA	Y	Y	Y	Y	NR	NA	NA	Poor
Moreau2012	PD	Y	Y	Y	Y	Y	Y	Y	Y	Y	Y	Y	N	Y	Y	Fair
Fremont2020	FTD	Y	NR	Y	Y	Y	Y	Y	Y	Y	Y	Y	NR	Y	Y	Good

AD = Alzheimer's disease, MCI = mild cognitive impairment, PD = Parkinson's disease, FTD = Frontotemporal dementia

Y = Yes; N = No; NA = not applicable; NR = not reported; CD = cannot determine.

Quality Rating: 1 in the 'No' column = Fair; >1 in the 'No' column = Poor. Consider Fair/Poor if too many questions cannot be answered. Questions were:

1. Was the study described as randomized, a randomized trial, a randomized clinical trial, or an RCT?
2. Was the method of randomization adequate (i.e., use of randomly generated assignment)?
3. Was the treatment allocation concealed (so that assignments could not be predicted)?
4. Were study participants and providers blinded to treatment group assignment?
5. Were the people assessing the outcomes blinded to the participants' group assignments?
6. Were the groups similar at baseline on important characteristics that could affect outcomes (e.g., demographics, risk factors, co-morbid conditions)?
7. Was the overall drop-out rate from the study at endpoint 20% or lower of the number allocated to treatment?
8. Was the differential drop-out rate (between treatment groups) at endpoint 15 percentage points or lower?

9. Was there high adherence to the intervention protocols for each treatment group?
10. Were other interventions avoided or similar in the groups (e.g., similar background treatments)?
11. Were outcomes assessed using valid and reliable measures, implemented consistently across all study participants?
12. Did the authors report that the sample size was sufficiently large to be able to detect a difference in the main outcome between groups with at least 80% power?
13. Were outcomes reported or subgroups analyzed prespecified (i.e., identified before analyses were conducted)?
14. Were all randomized participants analyzed in the group to which they were originally assigned, i.e., did they use an intention-to-treat analysis?

Supplementary Table S3. Checklist for meta-analysis of observational studies

Section/topic	#	Checklist item	Location Reported
**TITLE**			
Title	1	Identify the report as a systematic review, meta-analysis, or both.	Title
**ABSTRACT**			
Structured summary	2	Provide a structured summary including, as applicable: background; objectives; data sources; study eligibility criteria, participants, and interventions; study appraisal and synthesis methods; results; limitations; conclusions and implications of key findings; systematic review registration number.	Abstract
**INTRODUCTION**			
Rationale	3	Describe the rationale for the review in the context of what is already known.	Introduction
Objectives	4	Provide an explicit statement of questions being addressed with reference to participants, interventions, comparisons, outcomes, and study design (PICOS).	Introduction
**METHODS**			
Protocol and registration	5	Indicate if a review protocol exists, if and where it can be accessed (e.g. web address), and, if available, provide registration information including registration number.	PROSPERO CRD42021277500
Eligibility criteria	6	Specify study characteristics (e.g., PICOS, length of follow-up) and report characteristics (e.g., years considered, language, publication status) used as criteria for eligibility, giving rationale.	Methods
Information sources	7	Describe all information sources (e.g., databases with dates of coverage, contact with study authors to identify additional studies) in the search and date last searched.	Methods, Figure 1
Search	8	Present full electronic search strategy for at least one database, including any limits used, such that it could be repeated.	Table 2
Study selection	9	State the process for selecting studies (i.e., screening, eligibility, included in systematic review, and, if applicable, included in the meta-analysis).	Methods & Figure 1
Data collection process	10	Describe method of data extraction from reports (e.g., piloted forms, independently, in duplicate) and any processes for obtaining and confirming data from investigators.	Methods
Data items	11	List and define all variables for which data were sought (e.g., PICOS, funding sources) and any assumptions and simplifications made.	Methods & Table 1
Risk of bias in individual studies	12	Describe methods used for assessing risk of bias of individual studies (including specification of whether this was done at the study or outcome level), and how this information is to be used in any data synthesis.	Methods, results, Table S2
Summary measures	13	State the principal summary measures (e.g., risk ratio, difference in means).	Methods
Synthesis of results	14	Describe the methods of handling data and combining results of studies, if done, including measures of consistency (e.g., I^2) for each meta-analysis.	Methods, Results, Figures

Figure S1. Forest Plots of Noradrenergic Drugs on Global Cognition: Sub-analyses

A)

Study or Subgroup	Drug Mean	Drug SD	Drug Total	Placebo Mean	Placebo SD	Placebo Total	Weight	Std. Mean Difference IV, Random, 95% CI
Svenningsson2020	0.1	1.14	23	0.5	0.78	6	1.6%	−0.36 [−1.26, 0.54]
Herrmann2008	−0.58	2.53	13	−1.08	2.81	12	2.1%	0.18 [−0.61, 0.97]
Banerjee2021	4.6	10.1	23	−0.5	10.06	27	3.8%	0.50 [−0.07, 1.06]
Weintraub2010	0.12	1.53	28	−0.51	1.6	27	4.2%	0.40 [−0.14, 0.93]
Lanctot2014	0.27	9.24	25	0.6	7.6	30	4.2%	−0.04 [−0.57, 0.49]
Padala2018	5.7	9	30	−0.5	10	29	4.3%	0.64 [0.12, 1.17]
Fremont2020	3.89	23.65	28	5.21	23.14	28	4.3%	−0.06 [−0.58, 0.47]
Moreau2012	0	2.67	32	1.33	2.1	33	4.7%	−0.55 [−1.04, −0.05]
Levey2021	−1.71	3.19	35	−1.51	2.86	35	5.2%	−0.07 [−0.53, 0.40]
Mohs2009	1.2	3.39	46	0.7	3.35	45	6.3%	0.15 [−0.26, 0.56]
Maier2020	0.05	4.22	54	−0.4	4.16	54	7.1%	0.11 [−0.27, 0.48]
Mintzer2021	−1.4	3	83	−1	3	88	9.6%	−0.13 [−0.43, 0.17]
Huff1996a	−0.06	5.19	86	−1.33	4.93	90	9.7%	0.25 [−0.05, 0.55]
Huff1996b	−0.08	5.37	92	−1.33	4.93	90	9.9%	0.24 [−0.05, 0.53]
Amaducci1991	0.17	6.6	102	−1.38	6.6	95	10.4%	0.23 [−0.05, 0.51]
Winblad2001	0.19	6.93	148	−1	7.11	136	12.5%	0.17 [−0.06, 0.40]
**Total (95% CI)**			**848**			**825**	**100.0%**	**0.13 [0.01, 0.25]**

Heterogeneity: I^2 = 28%
Test for overall effect: Z = 2.05 (P = 0.04)

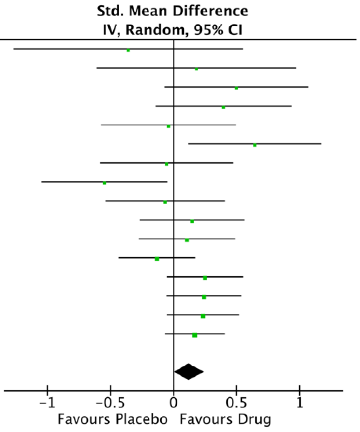

B)

Study	Drug Mean	Drug SD	Drug Total	Placebo Mean	Placebo SD	Placebo Total	Weight	Std. Mean Difference IV, Random, 95% CI
Moreau2012	0	2.67	32	1.33	2.1	33	38.0%	−0.55 [−1.04, −0.05]
Svenningsson2020	0.1	1.14	23	0.5	0.78	6	25.2%	−0.36 [−1.26, 0.54]
Weintraub2010	0.12	1.53	28	−0.51	1.6	27	36.7%	0.40 [−0.14, 0.93]
**Total (95% CI)**			**83**			**66**	**100.0%**	**−0.15 [−0.81, 0.50]**

Heterogeneity: I^2 = 70%
Test for overall effect: Z = 0.46 (P = 0.65)

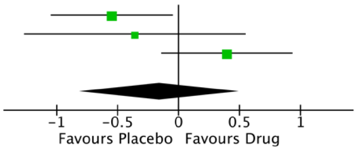

C)

Study	Drug Mean	Drug SD	Drug Total	Placebo Mean	Placebo SD	Placebo Total	Weight	Std. Mean Difference IV, Random, 95% CI
Herrmann2008	−0.58	2.53	13	−1.08	2.81	12	5.5%	0.18 [−0.61, 0.97]
Weintraub2010	0.12	1.53	28	−0.51	1.6	27	9.6%	0.40 [−0.14, 0.93]
Lanctot2014	0.27	9.24	25	0.6	7.6	30	9.6%	−0.04 [−0.57, 0.49]
Padala2018	5.7	9	30	−0.5	10	29	9.8%	0.64 [0.12, 1.17]
Moreau2012	0	2.67	32	1.33	2.1	33	10.5%	−0.55 [−1.04, −0.05]
Levey2021	−1.71	3.19	35	−1.51	2.86	35	11.2%	−0.07 [−0.53, 0.40]
Mohs2009	1.2	3.39	46	0.7	3.35	45	12.9%	0.15 [−0.26, 0.56]
Maier2020	0.05	4.22	54	−0.4	4.16	54	14.0%	0.11 [−0.27, 0.48]
Mintzer2021	−1.4	3	83	−1	3	88	16.9%	−0.13 [−0.43, 0.17]
**Total (95% CI)**			**346**			**353**	**100.0%**	**0.05 [−0.15, 0.26]**

Heterogeneity: I^2 = 44%
Test for overall effect: Z = 0.51 (P = 0.61)

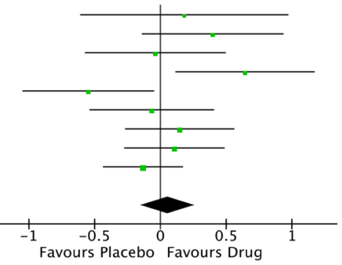

D)

Study	Drug Mean	Drug SD	Drug Total	Placebo Mean	Placebo SD	Placebo Total	Weight	Std. Mean Difference IV, Random, 95% CI
Svenningsson2020	0.1	1.14	23	0.5	0.78	6	5.0%	−0.36 [−1.26, 0.54]
Huff1996a	−0.06	5.19	86	−1.33	4.93	90	46.7%	0.25 [−0.05, 0.55]
Huff1996b	−0.08	5.37	92	−1.33	4.93	90	48.3%	0.24 [−0.05, 0.53]
**Total (95% CI)**			**201**			**186**	**100.0%**	**0.22 [0.01, 0.42]**

Heterogeneity: I^2 = 0%
Test for overall effect: Z = 2.08 (P = 0.04)

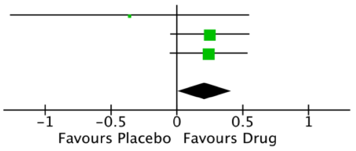

E)

Study	Drug Mean	Drug SD	Drug Total	Placebo Mean	Placebo SD	Placebo Total	Weight	Std. Mean Difference IV, Random, 95% CI
Herrmann2008	−0.58	2.53	13	−1.08	2.81	12	5.0%	0.18 [−0.61, 0.97]
Lanctot2014	0.27	9.24	25	0.6	7.6	30	10.4%	−0.04 [−0.57, 0.49]
Padala2018	5.7	9	30	−0.5	10	29	10.6%	0.64 [0.12, 1.17]
Levey2021	−1.71	3.19	35	−1.51	2.86	35	12.9%	−0.07 [−0.53, 0.40]
Mohs2009	1.2	3.39	46	0.7	3.35	45	16.1%	0.15 [−0.26, 0.56]
Maier2020	0.05	4.2	54	−0.4	4.16	54	18.6%	0.11 [−0.27, 0.48]
Mintzer2021	−1.4	3	83	−1	3	88	26.3%	−0.13 [−0.43, 0.17]
**Total (95% CI)**			**286**			**293**	**100.0%**	**0.07 [−0.11, 0.26]**

Heterogeneity: I^2 = 15%
Test for overall effect: Z = 0.80 (P = 0.43)

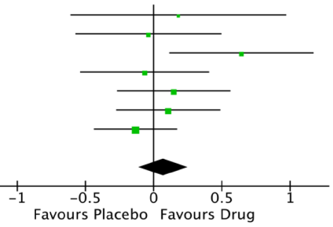

F)

Study	Drug Mean	Drug SD	Drug Total	Placebo Mean	Placebo SD	Placebo Total	Weight	Std. Mean Difference IV, Random, 95% CI
Banerjee2021	4.6	10.1	23	−0.5	10.06	27	9.1%	0.50 [−0.07, 1.06]
Amaducci1991	0.17	6.6	102	−1.38	6.6	95	37.2%	0.23 [−0.05, 0.51]
Winblad2001	0.19	6.93	148	−1	7.11	136	53.7%	0.17 [−0.06, 0.40]
**Total (95% CI)**			**273**			**258**	**100.0%**	**0.22 [0.05, 0.39]**

Heterogeneity: I^2 = 0%
Test for overall effect: Z = 2.56 (P = 0.01)

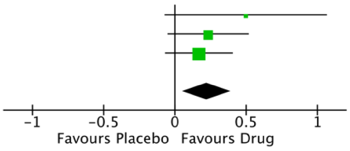

Figure S1. Comparison of drug and placebo for effect on global measures of cognition between baseline and end of treatment. IV = inverse variance; SD = standard deviation; CI = confidence interval.
A) All diagnoses; B) Parkinson's disease; C) All diagnoses – noradrenaline reuptake inhibitor; D) All diagnoses – alpha2 adrenergic receptor antagonist; E) Alzheimer's disease – noradrenaline reuptake inhibitor; F) Alzheimer's disease – alpha1 adrenergic receptor antagonist.

Figure S2. Forest Plots of Noradrenergic Drugs on Attention: Sub-analyses

A)

Study or Subgroup	Drug Mean	Drug SD	Drug Total	Placebo Mean	Placebo SD	Placebo Total	Weight	Std. Mean Difference IV, Random, 95% CI
Bedard1998	3.8	126.03	9	7.4	117.48	9	3.8%	−0.03 [−0.95, 0.90]
Lanctot2014	0.6	3	25	0.13	2.68	30	10.4%	0.16 [−0.37, 0.70]
Levey2021	−9.15	21.83	34	−3.94	15.43	34	12.5%	−0.27 [−0.75, 0.21]
Mintzer2021	0.1	1.7	82	0.1	1.5	84	25.1%	0.00 [−0.30, 0.30]
Mohr1989a	0.4	2.43	8	−0.4	2	8	3.3%	0.34 [−0.65, 1.33]
Mohr1989b	0.5	2.21	8	−0.4	2	8	3.3%	0.40 [−0.59, 1.40]
Mohr1989c	0.2	2.43	8	−0.4	2	8	3.3%	0.25 [−0.73, 1.24]
Moreau2012	27	120.9	33	−65	125.26	32	11.4%	0.74 [0.24, 1.24]
Rinne2017a	1	13.1	33	−0.7	9.2	34	12.4%	0.15 [−0.33, 0.63]
Rinne2017b	−3	13.2	33	−0.7	9.2	34	12.4%	−0.20 [−0.68, 0.28]
Schlegel1989	0.3	1.12	5	0	1.5	5	2.1%	0.20 [−1.04, 1.45]
**Total (95% CI)**			**278**			**286**	**100.0%**	**0.10 [−0.09, 0.28]**

Heterogeneity: $I^2 = 11\%$
Test for overall effect: Z = 1.04 (P = 0.30)

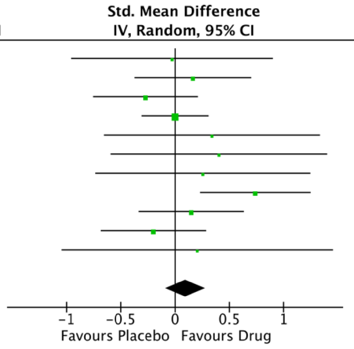

B)

Study or Subgroup	Drug Mean	Drug SD	Drug Total	Placebo Mean	Placebo SD	Placebo Total	Weight	Std. Mean Difference IV, Random, 95% CI
Lanctot2014	0.6	3	25	0.13	2.68	30	22.1%	0.16 [−0.37, 0.70]
Levey2021	−9.15	21.83	34	−3.94	15.43	34	24.0%	−0.27 [−0.75, 0.21]
Mintzer2021	0.1	1.7	82	0.1	1.5	84	30.8%	0.00 [−0.30, 0.30]
Moreau2012	27	120.9	33	−65	125.26	32	23.1%	0.74 [0.24, 1.24]
**Total (95% CI)**			**174**			**180**	**100.0%**	**0.14 [−0.24, 0.53]**

Heterogeneity: $I^2 = 67\%$
Test for overall effect: Z = 0.72 (P = 0.47)

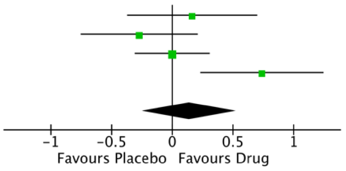

C)

Study or Subgroup	Drug Mean	Drug SD	Drug Total	Placebo Mean	Placebo SD	Placebo Total	Weight	Std. Mean Difference IV, Random, 95% CI
Lanctot2014	0.6	3	25	0.13	2.68	30	18.9%	0.16 [−0.37, 0.70]
Levey2021	−9.15	21.83	34	−3.94	15.43	34	23.4%	−0.27 [−0.75, 0.21]
Mintzer2021	0.1	1.7	82	0.1	1.5	84	57.7%	0.00 [−0.30, 0.30]
**Total (95% CI)**			**141**			**148**	**100.0%**	**−0.03 [−0.26, 0.20]**

Heterogeneity: $I^2 = 0\%$
Test for overall effect: Z = 0.28 (P = 0.78)

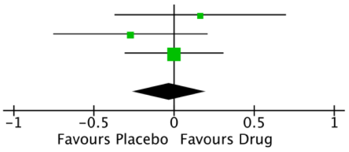

D)

Study or Subgroup	Drug Mean	Drug SD	Drug Total	Placebo Mean	Placebo SD	Placebo Total	Weight	Std. Mean Difference IV, Random, 95% CI
Mohr1989a	0.4	2.43	8	−0.4	2	8	27.5%	0.34 [−0.65, 1.33]
Mohr1989b	0.5	2.21	8	−0.4	2	8	27.3%	0.40 [−0.59, 1.40]
Mohr1989c	0.2	2.43	8	−0.4	2	8	27.8%	0.25 [−0.73, 1.24]
Schlegel1989	0.3	1.12	5	0	1.5	5	17.4%	0.20 [−1.04, 1.45]
**Total (95% CI)**			**29**			**29**	**100.0%**	**0.31 [−0.21, 0.83]**

Heterogeneity: $I^2 = 0\%$
Test for overall effect: Z = 1.17 (P = 0.24)

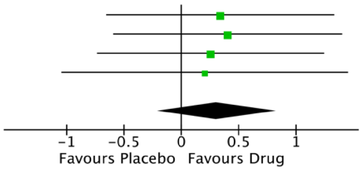

Figure S2. Comparison of drug and placebo for effect on measures of attention between baseline and end of treatment. IV = inverse variance; SD = standard deviation; CI = confidence interval.
A) All diagnoses; B) All diagnoses – noradrenaline reuptake inhibitor; C) Alzheimer's disease – noradrenaline reuptake inhibitor; D) Alzheimer's disease – alpha2 adrenergic receptor agonist.

Figure S3. Forest Plots of Noradrenergic Drugs on Cognitive Sub-Domains: Sub-analyses

A)

Study or Subgroup	Drug Mean	Drug SD	Drug Total	Placebo Mean	Placebo SD	Placebo Total	Weight	Std. Mean Difference IV, Random, 95% CI
Bedard1998	59.1	86.96	9	−23.4	128.12	9	5.3%	0.72 [−0.24, 1.68]
Crook1992	−1.93	4.01	15	−0.46	2.7	14	7.6%	−0.42 [−1.15, 0.32]
Lanctot2014	0.32	2.51	25	−0.13	2.11	30	11.0%	0.19 [−0.34, 0.72]
Levey2021	−29.03	52.68	33	5.34	74.22	33	11.8%	−0.53 [−1.02, −0.04]
Mintzer2021	0.3	1.5	82	−0.2	1.9	84	16.1%	0.29 [−0.02, 0.60]
Mohr1989a	−0.4	1.9	8	0.2	1.2	8	5.0%	−0.36 [−1.35, 0.63]
Mohr1989b	−0.7	1.9	8	0.2	1.2	8	4.9%	−0.54 [−1.54, 0.47]
Mohr1989c	−0.1	1.65	8	0.2	1.2	8	5.1%	−0.20 [−1.18, 0.79]
Rinne2017a	0.2	0.63	33	−0.21	0.56	34	11.8%	0.68 [0.19, 1.17]
Rinne2017b	−0.02	0.62	33	−0.21	0.56	34	12.0%	0.32 [−0.16, 0.80]
Schlegel1989	0.6	1.7	5	0.4	1.63	5	3.5%	0.11 [−1.13, 1.35]
Svenningsson2020	0.6	12.4	26	−2.8	11.02	6	5.9%	0.27 [−0.62, 1.16]
**Total (95% CI)**			**285**			**273**	**100.0%**	**0.10 [−0.16, 0.35]**

Heterogeneity: I^2 = 45%
Test for overall effect: Z = 0.74 (P = 0.46)

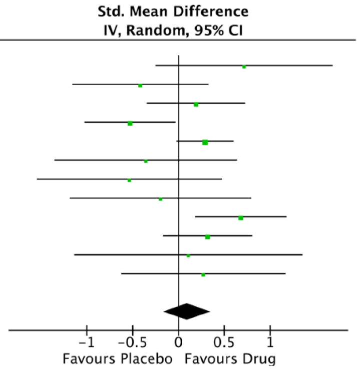

B)

Study or Subgroup	Drug Mean	Drug SD	Drug Total	Placebo Mean	Placebo SD	Placebo Total	Weight	Std. Mean Difference IV, Random, 95% CI
Bedard1998	5.8	9.26	9	3.3	10.07	9	4.1%	0.25 [−0.68, 1.17]
Levey2021	−1.08	3.21	35	−0.68	4.14	35	16.0%	−0.11 [−0.58, 0.36]
Mintzer2021	0.8	3.5	78	−0.3	2.8	84	36.5%	0.35 [0.04, 0.66]
Mohr1989a	1.2	3.62	8	−0.8	5.82	8	3.6%	0.39 [−0.60, 1.38]
Mohr1989b	2.4	4.42	8	−0.8	5.82	8	3.5%	0.59 [−0.42, 1.59]
Mohr1989c	1.4	3.62	8	−0.8	5.82	8	3.6%	0.43 [−0.57, 1.42]
Rinne2017a	0.1	3.2	33	0.6	3.1	34	15.3%	−0.16 [−0.64, 0.32]
Rinne2017b	1.6	2.6	33	0.6	3.1	34	15.1%	0.35 [−0.14, 0.83]
Schlegel1989	1	6.5	5	0.2	6.36	5	2.3%	0.11 [−1.13, 1.35]
**Total (95% CI)**			**217**			**225**	**100.0%**	**0.20 [0.01, 0.39]**

Heterogeneity: I^2 = 0%
Test for overall effect: Z = 2.09 (P = 0.04)

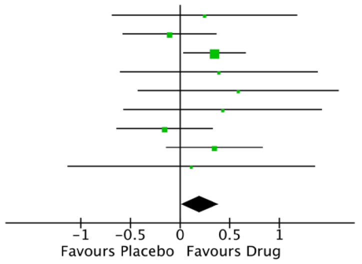

C)

Study or Subgroup	Drug Mean	Drug SD	Drug Total	Placebo Mean	Placebo SD	Placebo Total	Weight	Std. Mean Difference IV, Random, 95% CI
Bedard1998	2.8	4.25	9	2	4.86	9	17.2%	0.17 [−0.76, 1.09]
Crook1992	−1.53	2.04	15	−0.54	2.16	14	27.0%	−0.46 [−1.20, 0.28]
Mohr1989a	1	2.83	8	0.7	4.05	8	15.4%	0.08 [−0.90, 1.06]
Mohr1989b	0.5	2.83	8	0.7	4.05	8	15.4%	−0.05 [−1.03, 0.93]
Mohr1989c	0.5	3.06	8	0.7	4.05	8	15.4%	−0.05 [−1.03, 0.93]
Schlegel1989	−0.7	2.98	5	−0.5	3.11	5	9.6%	−0.06 [−1.30, 1.18]
**Total (95% CI)**			**53**			**52**	**100.0%**	**−0.10 [−0.49, 0.28]**

Heterogeneity: I^2 = 0%
Test for overall effect: Z = 0.53 (P = 0.59)

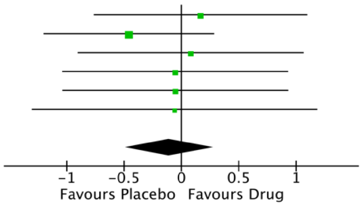

Figure S3. Comparison of drug and placebo for effect on measures of cognitive sub-domains between baseline and end of treatment, across all diagnoses. IV = inverse variance; SD = standard deviation; CI = confidence interval.
A) Executive functions and working memory; B) Semantic memory; C) Visuospatial abilities.

Figure S4. Forest Plots of Noradrenergic Drugs on Neuropsychiatric Symptoms: Sub-analyses

A)

Study or Subgroup	Drug Mean	Drug SD	Drug Total	Placebo Mean	Placebo SD	Placebo Total	Weight	Std. Mean Difference IV, Random, 95% CI
Banerjee2021	−8.8	17.8	75	−9.2	20.06	84	18.7%	0.02 [−0.29, 0.33]
Fremont2020	0.93	6.55	28	0.46	6.78	28	8.8%	0.07 [−0.45, 0.59]
Huff1996a	−0.55	3.73	87	−0.24	3.74	92	20.2%	−0.08 [−0.38, 0.21]
Huff1996b	−0.24	3.74	92	−0.24	3.74	92	20.5%	0.00 [−0.29, 0.29]
Levey2021	−4.6	6.22	35	−3.86	9.53	35	10.5%	−0.09 [−0.56, 0.38]
Maier2020	−0.24	10.5	54	−5.75	10.25	54	14.2%	0.53 [0.14, 0.91]
Peskind2005	8	21.03	17	0.4	22.28	13	5.0%	0.34 [−0.39, 1.07]
Wang2009	19	21	7	2	15	6	2.1%	0.85 [−0.31, 2.01]
**Total (95% CI)**			**395**			**404**	**100.0%**	**0.09 [−0.08, 0.27]**

Heterogeneity: $I^2 = 27\%$
Test for overall effect: Z = 1.07 (P = 0.28)

Std. Mean Difference IV, Random, 95% CI

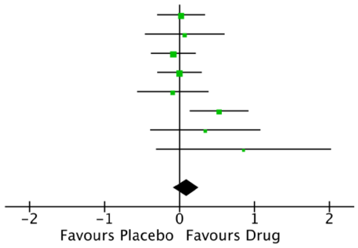

B)

Study or Subgroup	Drug Mean	Drug SD	Drug Total	Placebo Mean	Placebo SD	Placebo Total	Weight	Std. Mean Difference IV, Random, 95% CI
Frakey2012	6.55	14.45	11	6.82	18.04	11	7.6%	−0.02 [−0.85, 0.82]
Herrmann2008	2.31	5.11	13	−0.5	3.87	12	8.0%	0.60 [−0.21, 1.40]
Levey2021	−0.17	2.31	35	−0.63	2.41	35	13.7%	0.19 [−0.28, 0.66]
Maier2020	0.14	8.23	54	−2.07	8	54	15.8%	0.27 [−0.11, 0.65]
Mintzer2021	4.46	4.12	89	3.11	3.64	91	17.8%	0.35 [0.05, 0.64]
Moreau2012	−0.33	7.3	33	−1	7.41	32	13.4%	0.09 [−0.40, 0.58]
Padala2018	14.1	7.1	30	4.2	5.7	29	11.4%	1.51 [0.93, 2.10]
Weintraub2010	1.91	4.42	28	0.1	4.6	27	12.4%	0.40 [−0.14, 0.93]
**Total (95% CI)**			**293**			**291**	**100.0%**	**0.41 [0.13, 0.69]**

Heterogeneity: $I^2 = 61\%$
Test for overall effect: Z = 2.83 (P = 0.005)

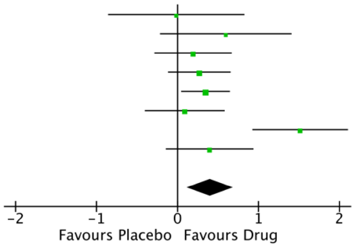

C)

Study or Subgroup	Drug Mean	Drug SD	Drug Total	Placebo Mean	Placebo SD	Placebo Total	Weight	Std. Mean Difference IV, Random, 95% CI
Frakey2012	6.55	14.45	11	6.82	18.04	11	11.2%	−0.02 [−0.85, 0.82]
Herrmann2008	2.31	5.11	13	−0.5	3.87	12	11.7%	0.60 [−0.21, 1.40]
Levey2021	−0.17	2.31	35	−0.63	2.41	35	18.4%	0.19 [−0.28, 0.66]
Maier2020	0.14	8.23	54	−2.07	8	54	20.5%	0.27 [−0.11, 0.65]
Mintzer2021	4.46	4.12	89	3.11	3.64	91	22.3%	0.35 [0.05, 0.64]
Padala2018	14.1	7.1	30	4.2	5.7	29	15.9%	1.51 [0.93, 2.10]
**Total (95% CI)**			**232**			**232**	**100.0%**	**0.48 [0.10, 0.85]**

Heterogeneity: $I^2 = 70\%$
Test for overall effect: Z = 2.51 (P = 0.01)

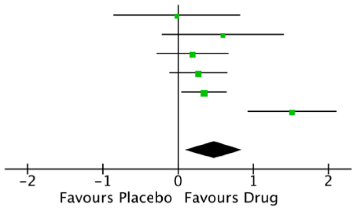

Figure S4. Comparison of drug and placebo for effect on measures of neuropsychiatric symptoms between baseline and end of treatment. IV = inverse variance; SD = standard deviation; CI = confidence interval.
A) General neuropsychiatric symptoms all diagnoses; B) Apathy all diagnoses – noradrenaline reuptake inhibitor; C) Apathy Alzheimer's disease – noradrenaline reuptake inhibitor.

Figure S5. Funnel Plots of All Studies of Noradrenergic Drugs in Neurodegenerative Conditions

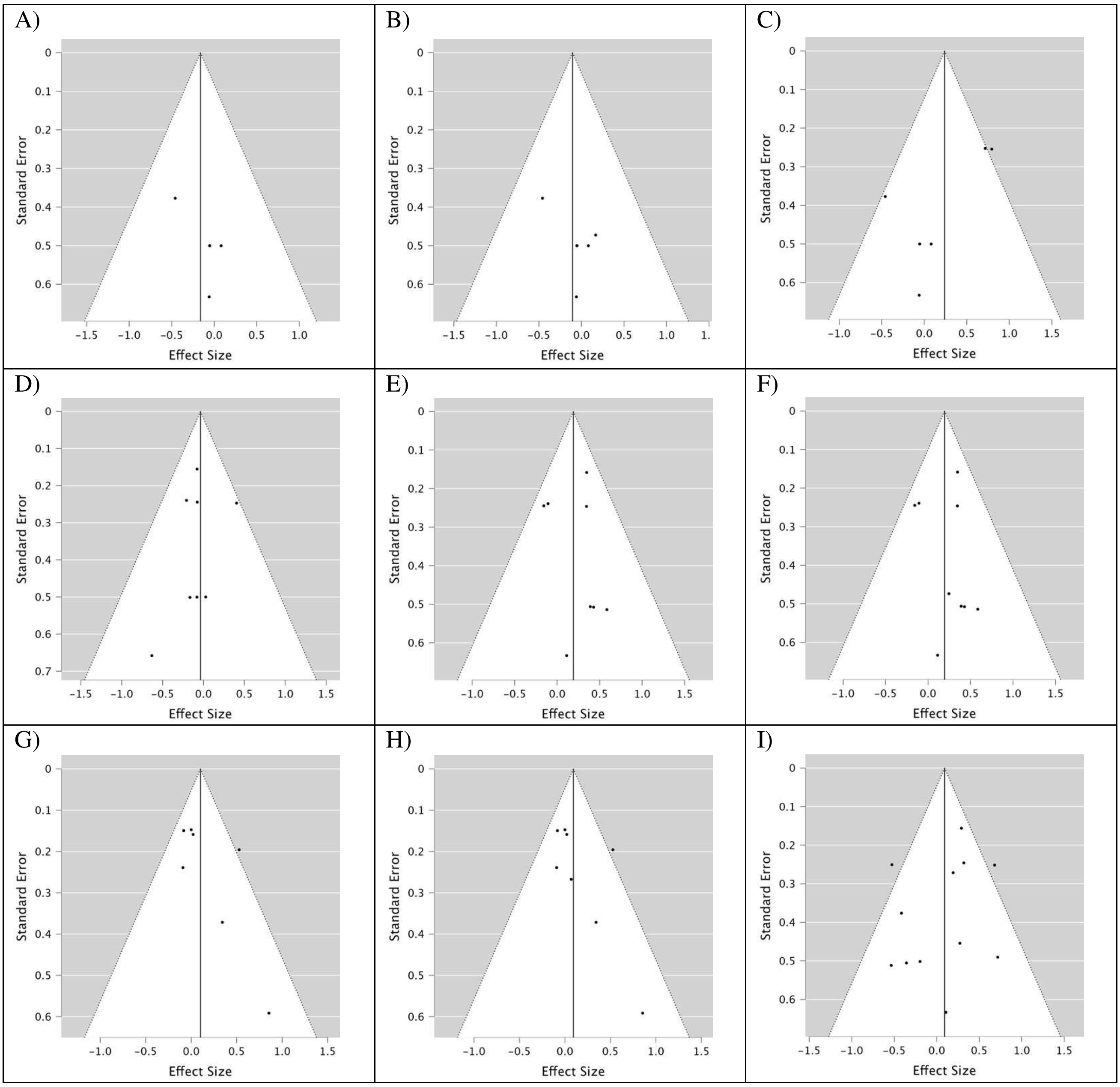

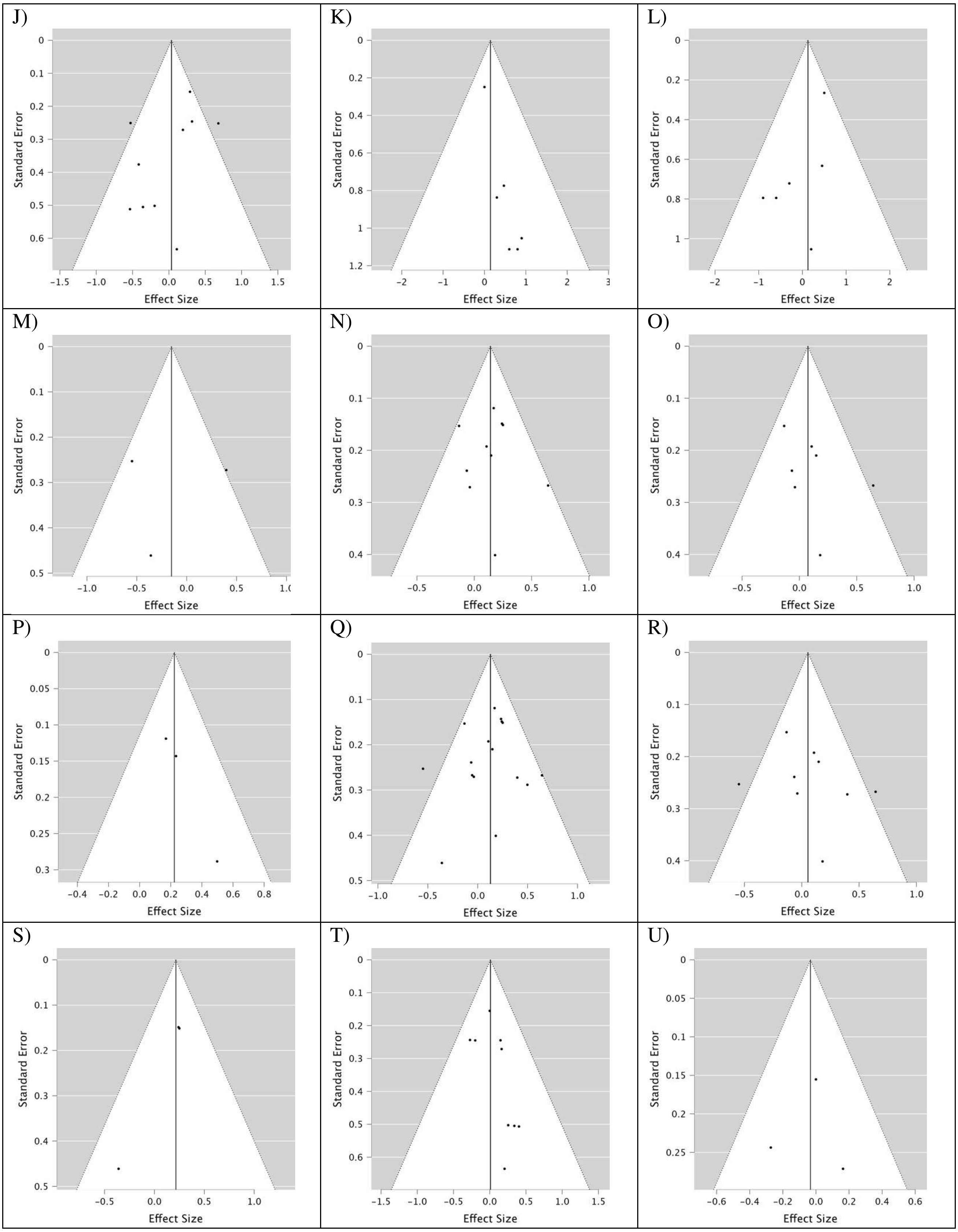
J)
K)
L)
M)
N)
O)
P)
Q)
R)
S)
T)
U)
Standard Error
Effect Size

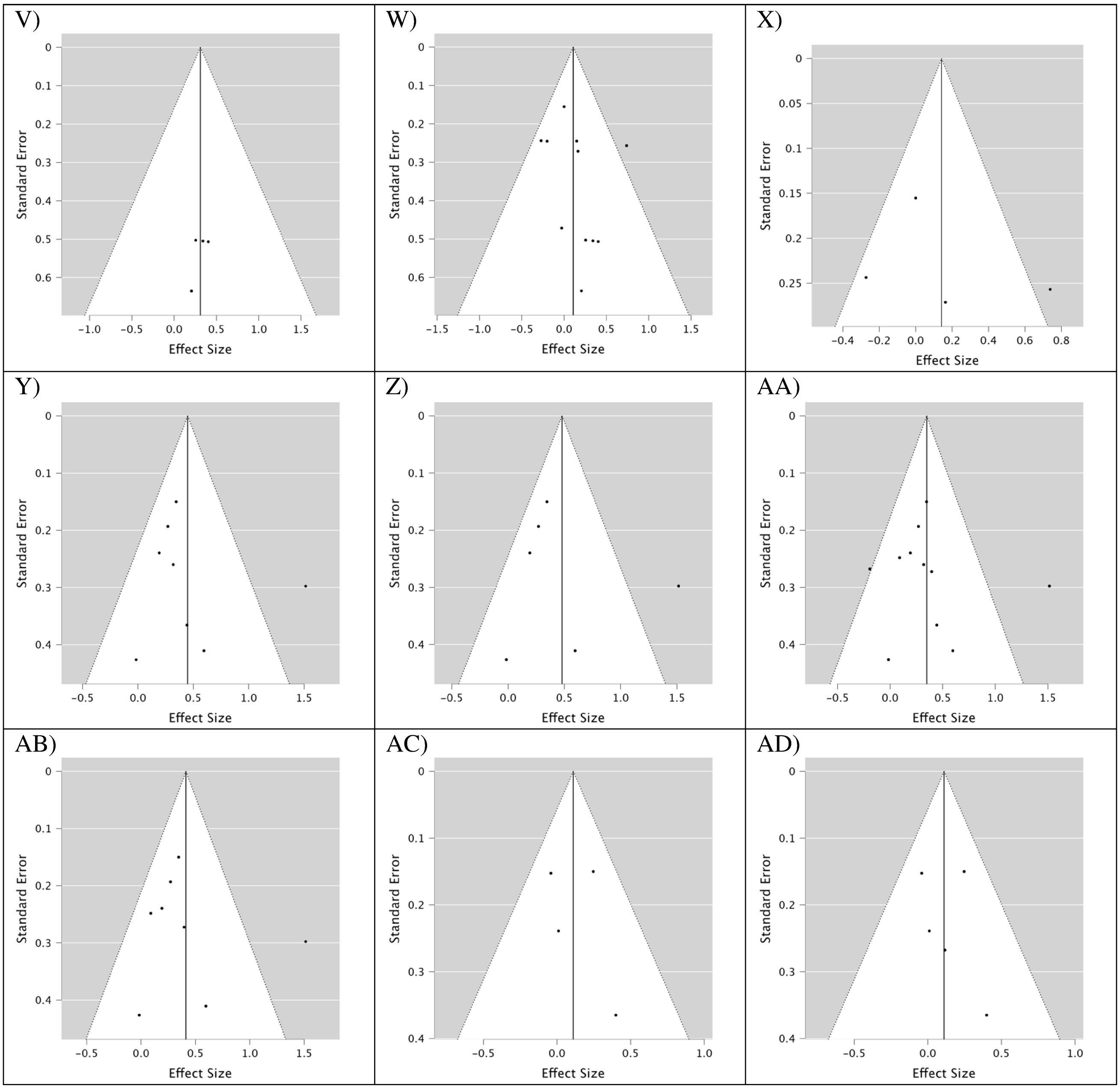

Figure S5. Funnel Plots to identify asymmetry that may be indicative of publication bias.
A) Visuospatial abilities in AD; B) Visuospatial abilities all diagnoses; C) Visual episodic memory in AD; D) Verbal episodic memory in AD; E) Semantic memory in AD; F) Semantic memory all diagnoses; G) General neuropsychiatric symptoms in AD; H) General neuropsychiatric symptoms all diagnoses; I) Executive functions and working memory all diagnoses; J) Executive functions and working memory in AD; K) Digit span forwards in AD; L) Digit span backwards in AD; M) Global cognition in PD; N) Global cognition in AD; O) Global cognition in AD – NRI; P) Global cognition in AD – A1 Ant; Q) Global cognition all diagnoses; R) Global cognition all diagnoses – NRI; S) Global cognition all diagnoses – A2 Ant; T) Attention in AD; U) Attention in AD – NRI; V) Attention in AD – A2 Ag; W) Attention all diagnoses; X) Attention all diagnoses – NRI; Y) Apathy in AD; Z) Apathy in AD – NRI; AA) Apathy all diagnoses; AB) Apathy all diagnoses – NRI; AC) Agitation in AD; AD) Agitation all diagnoses.

AD = Alzheimer's disease, PD = Parkinson's disease, NRI = Noradrenaline reuptake inhibitor, A1 Ant = alpha1 adrenergic receptor antagonist, A2 Ag = alpha2 adrenergic receptor agonist, B Ant = Beta adrenergic receptor antagonist/blocker, A2 Ant = alpha2 adrenergic receptor antagonist.

Table S4. Results of Meta-Regression on Global Cognition Analysis				
**Covariates**	**Number of studies**	**β (CI 95%)**	**P value**	**Proportion of variance explained**
Age	8	0.0224 (-0.0735, 0.1183)	0.5886	0.00%
Gender (% female)	8	-0.0044 (-0.0149, 0.0061)	0.3459	0.00%
Duration of treatment (weeks)	10	-0.0095 (-0.0259, 0.0069)	0.2186	0.00%
Year of publication	10	-0.0011(-0.0994, 0.0972)	0.9793	0.00%

Table S5. Results of Meta-Regression on Apathy Analysis				
**Covariate**	**Number of studies**	**β (CI 95%)**	**P value**	**Proportion of variance explained**
Age	8	0.0213 (-0.0911, 0.1337)	0.6597	0.00%
Gender (% female)	7	-0.025 (-0.0289, 0.0040)	0.1091	53.41%
Duration of treatment (weeks)	8	-0.0080 (-0.0551, 0.0391)	0.6921	0.00%
Year of publication	8	-0.0011 (-0.0994, 0.0972)	0.9793	0.00%

Figure S6. Bubble Plots for Meta-Regression of Effect of Covariates on Results of Global Cognition and Apathy Meta-Analyses

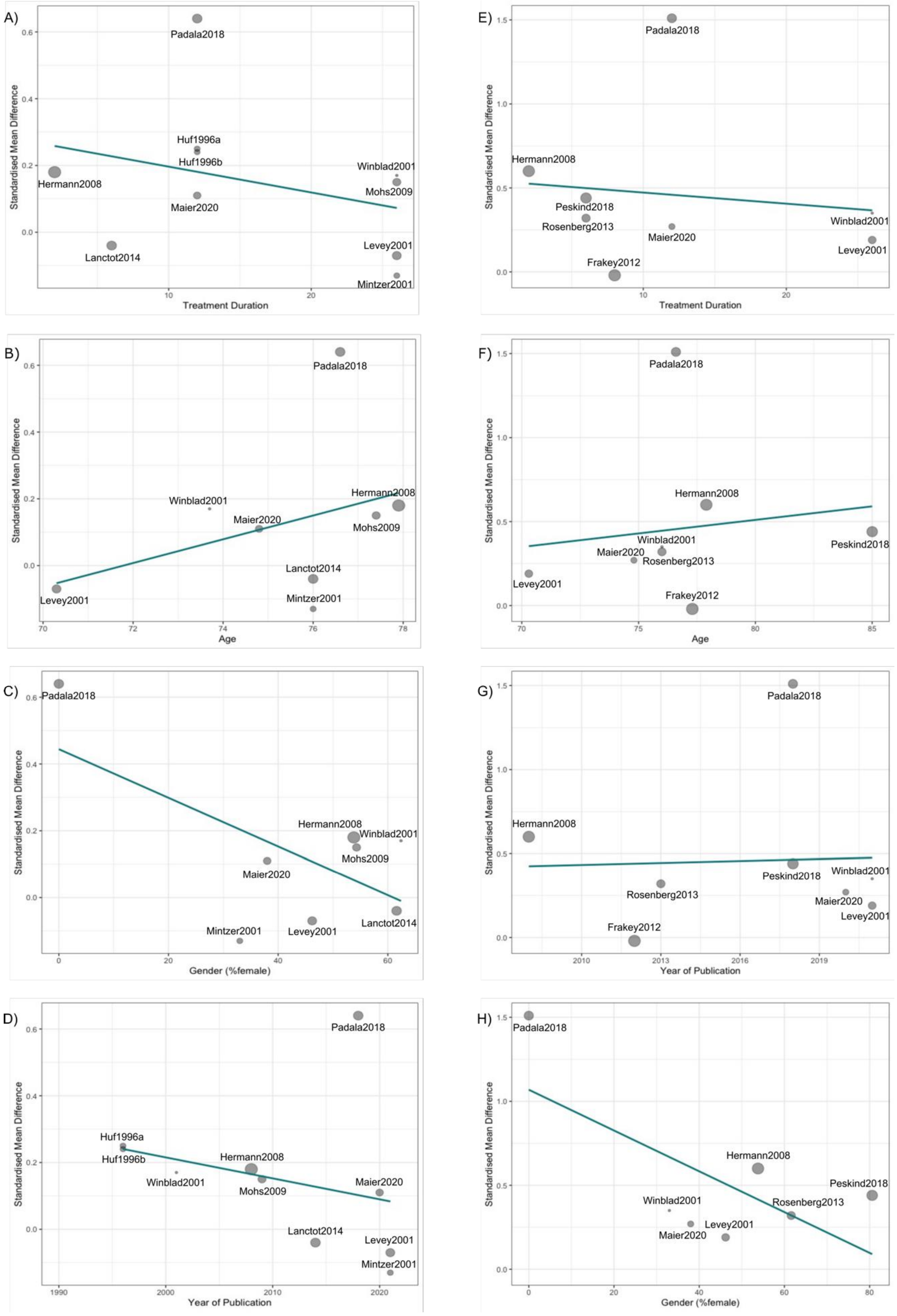

Figure S6. Bubble plots to investigate if covariates were significantly associated with effect size differences. Bubble size represents standard error. Plots A)-D) are for global cognition analysis, plots E)-H) are for apathy.

References

[1] Weintraub D, Mavandadi S, Mamikonyan E, Siderowf AD, Duda JE, Hurtig HI, et al. Atomoxetine for depression and other neuropsychiatric symptoms in Parkinson disease. Neurology 2010;75:448–55. https://doi.org/10.1212/WNL.0b013e3181ebdd79.

[2] Moreau C, Delval A, Defebvre L, Dujardin K, Duhamel A, Petyt G, et al. Methylphenidate for gait hypokinesia and freezing in patients with Parkinson's disease undergoing subthalamic stimulation: A multicentre, parallel, randomised, placebo-controlled trial. Lancet Neurol 2012;11:589–96. https://doi.org/10.1016/S1474-4422(12)70106-0.

[3] Bédard MA, El Massioui F, Malapani C, Dubois B, Pillon B, Renault B, et al. Attentional deficits in Parkinson's disease: Partial reversibility with naphtoxazine (SDZ NVI-085), a selective noradrenergic α1 agonist. Clin Neuropharmacol 1998;21:108–17.

[4] Svenningsson P, Odin P, Dizdar N, Johansson A, Grigoriou S, Tsitsi P, et al. A Phase 2a Trial Investigating the Safety and Tolerability of the Novel Cortical Enhancer IRL752 in Parkinson's Disease Dementia. Mov Disord 2020;35:1046–54. https://doi.org/10.1002/mds.28020.

[5] Fremont R, Manoochehri M, Armstrong NM, Mattay VS, Apud JA, Tierney MC, et al. Tolcapone Treatment for Cognitive and Behavioral Symptoms in Behavioral Variant Frontotemporal Dementia: A Placebo-Controlled Crossover Study. J Alzheimer's Dis 2020;75:1437–46. https://doi.org/10.3233/JAD-191265.

[6] Wan X, Wang W, Liu J, Tong T. Estimating the sample mean and standard deviation from the sample size, median, range and/or interquartile range. BMC Med Res Methodol 2014;14:1–13. https://doi.org/10.1186/1471-2288-14-135.

[7] Higgins JPT, Tianjing Li, Deeks JJ,. Chapter 6: Choosing effect measures and computing estimates of effect. Cochrane Handb. Syst. Rev. Interv. version 6.2, vol. 1, Cochrane; 2020. n.d.
